# Integrating population genomics and environmental data to predict adaptation to climate change in post-bottleneck Tibetan macaques

**DOI:** 10.1126/sciadv.adw0562

**Published:** 2025-07-09

**Authors:** Yang Teng, Wenbo Li, Xiaochen Wang, Rusong Zhang, Ying Shen, Ruifeng Wu, Jiawen Liu, Mingyi Zhang, Christian Roos, Jinhua Li, Jing Li, Jiwei Qi, Ming Li

**Affiliations:** ^1^ State Key Laboratory of Animal Biodiversity Conservation and Integrated Pest Management (Key Laboratory of Animal Ecology and Conservation Biology), Institute of Zoology, Chinese Academy of Sciences, Beijing 100101, China.; ^2^ University of Chinese Academy of Sciences, Beijing 100049, China.; ^3^ International Collaborative Research Center for Huangshan Biodiversity and Tibetan Macaque Behavioral Ecology, Anhui University, Hefei, Anhui 230601, China.; ^4^ Key Laboratory of Bio-resources and Eco-environment (Ministry of Education), College of Life Sciences, Sichuan University, Chengdu 610065, PR China.; ^5^ Yunnan Institute of Forest Inventory and Planning, Kunming 650000, PR China.; ^6^ Primate Genetics Laboratory, German Primate Center, Leibniz Institute for Primate Research, Kellnerweg 4, 37077 Göttingen, Germany.; ^7^ Hefei Normal University/International Collaborative Research Center for Huangshan Biodiversity and Tibetan Macaque Behavioral Ecology, Hefei, Anhui, China.

## Abstract

Rapid climate change represents a profound threat to biodiversity. Understanding the local adaptations and their vulnerabilities to climate change are imperative for developing conservation measures. Here, we combined a multidisciplinary approach to determine the local adaptations of an endemic and near-threatened primate, aiming to reveal its potential to cope with future climate change. Results suggest that climatic fluctuations played an important role in shaping its demographic trajectory and genetic structure. In addition, Tibetan macaques have experienced a severe bottleneck in the recent past, with highly deleterious mutations partially removed, but moderately deleterious mutations accumulating. The severe bottleneck and lower genetic diversity may have reduced their potential to adapt to environmental change, which will compromise long-term viability. Furthermore, we found that the eastern group exhibited higher genomic offsets and loss of suitable habitat in response to climate change. Overall, we emphasize the importance of integrating population genomics and environmental data to predict the adaptation of post-bottleneck populations to rapid climate change.

## Teaser

Genomics and environmental data were used to quantify how recent population bottlenecks have eroded adaptive potential.

## INTRODUCTION

As the global climate deviates from its historical norm, species are increasingly exposed to conditions beyond their physiological tolerance (*1*, *2*). This forces them to find suitable locations or adapt to changing environments through adaptive evolution to avoid population collapse (*3*). Consequently, it is crucial to comprehend and quantify the local adaptation and climate change–driven vulnerability of a species. This is not only relevant for understanding whether and how species can survive in a changing climate, but can enhance conservation and management strategies to cope with the loss of global biodiversity (*4*, *5*).

Climate change is recognized as a major threat to global biodiversity (*6*). However, what do we actually know about how climate change causes extinction? Numerous studies have shown that limited physiological tolerances to high temperatures should be the major factor that causes climate change to threaten the persistence of populations and species (*7*, *8*). Studies have shown that increased temperatures can push ectothermic animals beyond their physiological tolerance limits, affecting their metabolism, growth, reproduction, and survival (*9*, *10*); however, while endothermic animals can maintain relatively constant body temperatures, they also face challenges such as high temperatures that increase their energy expenditure to regulate their body temperatures, which can lead to decreased reproductive success and reduced population size (*11*). In addition, for example, negative impacts of heat-avoidance behavior (*12*), the climate-related loss of host and pollinator species (*13*), positive impacts of climate change on pathogens and competitors (*14*), and other factors can also reduce long-term viability. When they survive, genetic drift and inbreeding may reduce the fitness of individuals and the survival potential of the population (*15*). Bottlenecks influence the ability of populations to adapt to a challenging future environment in two major ways (*16*). First, recent bottlenecks can result in a substantial loss of genetic diversity, whereas selection is weaker relative to genetic drift when the population size is small. Genetic drift might alter the genomic landscape during bottlenecks, resulting in a decline in population fitness (*17*). Second, after bottleneck events, inbreeding can increase because of the higher homozygosity of alleles that are common by descent (*16*, *18*). When homozygosity increases at loci with deleterious alleles, inbreeding leads to inbreeding depression (*19*–*21*). Thus, examining the evolutionary genomic consequence of recovering populations after bottlenecks can improve our understanding of the viability, recovery potential, and long-term adaptive capacity of threatened populations.

Local populations may need to quickly adapt to the challenges driven by climate change to maintain population stability (*22*); however, local populations have to prevent a reduction in long-term viability through a regional shift when the extent of climate change exceeds their ability to tolerate at their current location (*23*). If populations that are not adapted to the current climate have climate-adapted genotypes (also known as “preadapted”), suggesting that they may have already adapted to the environment in the shift region, then future maladaptation can be minimized (*24*). Studies indicate that genome scans and genotype-environment associations (GEA) can be done for genomic predictions of climate adaptation (*22*, *25*). The association of climate-adaptive candidate genes with the environment may be projected through time to evaluate mismatches in their predicted distributions across present and future landscapes, which is known as genomic offset or genomic vulnerability (*26*). These evaluations to some extent exclude the possibility of phenotypic plasticity or future adaptation to mitigate susceptibility. Instead, they presume that candidate loci have causal impacts on fitness, which is difficult to verify (*27*). Furthermore, the assumption of monotonic cline between allele frequency and environment may not hold for most alleles involved in climate adaptation, as associations between environment and genomic variation in highly variable organisms have been considered to be highly polygenic, limiting the effectiveness of GEA methods in detecting actual causal loci (*28*). Therefore, there is a growing need for more sophisticated approaches that explicitly consider and model the genomic complexity of polygenic adaptations to reveal the genetic structure of species adapting to their environments (*29*). However, GEA still provides a useful tool for determining key environmental drivers of climate change risk. For ecologically important taxa with limited tractability for experimentation, it may be useful to identify areas that are at risk of decline if they are unable to adapt in situ to future climates (*27*, *30*).

Nonhuman primates are very sensitive to small changes in temperature. Because of their nonmigratory and long generation times, among other characteristics, they are expected to be among the mammals that will be proportionately more adversely affected by recent climate change (*31*). In addition, nonhuman primates are highly sensitive to climate variability and change, especially changes in temperature and precipitation (*31*, *32*). Studies indicate that changes in these climatic variables have a negative effect on primate behavior, diet, and reproductive rates (*33*). For example, Ozgul *et al.* (*34*) found that decreasing rainfall and increasing seasonal temperatures disrupted population persistence in the gray mouse lemur (*Microcebus murinus*), a small primate with a fast life history. This suggests that primates are being threatened by the effect of climate change on demographic mechanisms. The Tibetan macaque (*Macaca thibetana*) is a primate endemic to China. It is primarily distributed in the hilly areas of southeastern China and the mountainous areas of southwestern China (*35*). There are large climatic variations [two climatic zones: subtropical and highland climatic zones; four climate types: dry winter humid subtropical climate (Cwa), dry winter subtropical highland climate (Cwb), humid subtropical climate (Cfa), oceanic climate (Cfb), and a maximum mean annual temperature difference of 23°C] within its distribution (*36*), indicating that this species is a suitable model for exploring genetic adaptations to varying climates. Recently, Li *et al.* (*35*) predicted potentially suitable habitats and possible migration directions of Tibetan macaques facing future climate change, finding that Tibetan macaques may face an increased risk of extinction because of future climate change. Therefore, understanding a species’ local adaptation and its climate change–driven vulnerability played an important role in uncovering whether and how species can survive under the present and future climate changes (*5*), which can now be addressed by incorporating intraspecific genomic variation in modeling habitat suitability to understand fine-scale estimates of climate change–driven vulnerability (*37*). In addition, some studies show that Tibetan macaques can be classified into four genetically different groups (*38*). There was a historical population bottleneck and an ancient introgression with the rhesus macaque (*Macaca mulatta*) during its evolutionary process (*39*). Recent studies have shown that as most hybridizing species are likely to differ in their demographic histories and population sizes, the pattern and magnitude of deleterious variations and genetic load can vary dramatically among these species (*40*). When there is gene flow from a donor species with a smaller effective population size and reduced efficiency of purifying selection, the recipient species may suffer from increased genetic load owing to the introduction of weakly deleterious alleles (*41*). In contrast, gene flow from a donor species with larger effective population size could alleviate the genetic load of the recipient species (*42*). Therefore, selection on deleterious introgressed variants could have a profound impact on the genome-wide patterns of introgression (*43*). In addition, although hybridization between divergently adapted species is largely deleterious, introgression can occasionally introduce adaptive variants that are favored by positive selection and can spread rapidly in the recipient species, a process known as adaptive introgression (*44*). Introgression, including adaptive introgression, is increasingly being recognized as an important source of diversity and adaptation across taxa (*45*). However, it is still unclear whether and how this species can adapt to a local environment and respond to climate change after bottleneck. This is necessary for creating effective conservation and management plans for this species, especially for some post-bottleneck populations to gain insight into whether and how they can survive under future climatic circumstances.

**Fig. 1.**
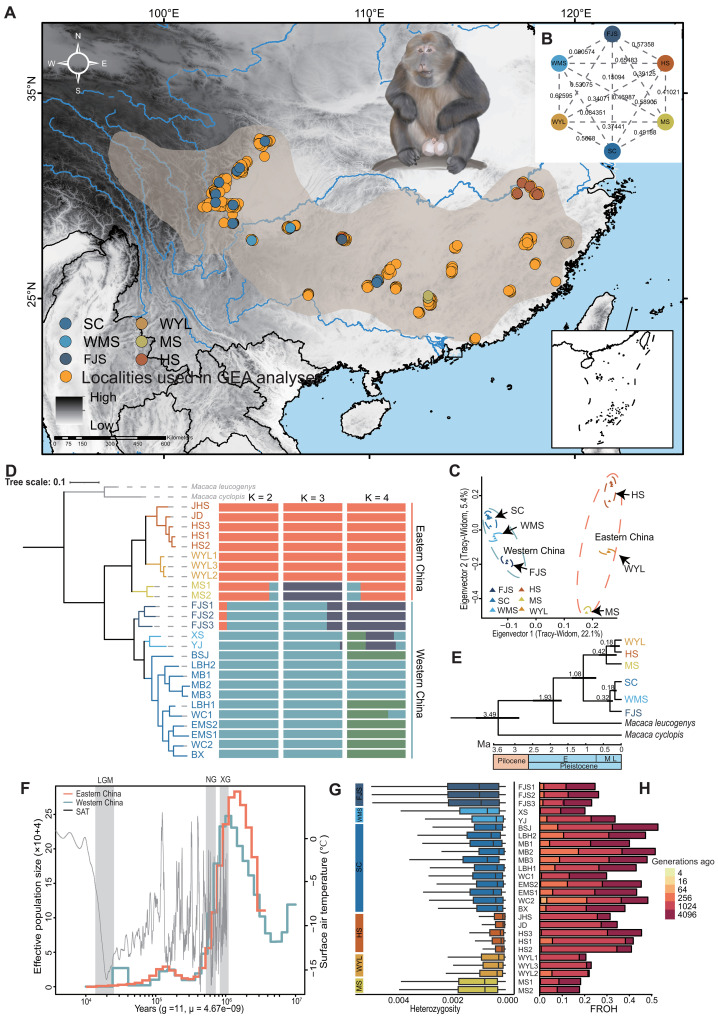
Distribution of locations of Tibetan macaques and estimates of genetic structure, demographic history and divergence time, heterozygosity, and inbreeding level. (**A**) Sample locations. The blue outlines are the possible ranges of the species, the yellow circles indicate the exact species distribution loci obtained from the field surveys, and the other colored circles indicate the genomic samples collected from the various populations. (**B**) Pairwise *Fst* values between populations, with lower *Fst* values within eastern and western populations, and lower *Fst* values between eastern and western populations. (**C**) PCA results based on whole-genome SNPs. (**D**) Maximum likelihood phylogenetic tree based on whole-genome SNPs, with *Macaca leucogenys* and *Macaca cyclopis* as the outgroup. Admixture results based on whole-genome SNPs with *K* = 2 to 4 (**E**) Estimation of divergence time. Black bars indicate 95% confidence intervals of divergence times, and the time scale below shows million years ago (Ma). Abbreviations: L, late; M, middle; E, early. Two fossil-based calibration points are as follows: *M. cyclopis* 3.3 to 4.3 Ma, *M. leucogenys* 1.8 to 2.4 Ma. (**F**) PSMC analysis revealed demographic histories of the two populations, with a generation time (g) of 11 years and a mutation rate (μ) of 4.67 × 10^−09^ per site per generation. The time axis is logarithmic transformed. The periods of the Xixiabangma Glaciation (XG, 0.8 to 1.17 ka), Naynayxungla Glaciation (NG, 0.5 to 0.78 ka), last glacial maximum (LGM, 12 to 30 ka) are shaded in gray. SAT, surface air temperature relative to the present. (**G**) Individual genome-wide heterozygosity. (**H**) Partitioning of the genome in various HBD classes in Tibetan Macaque populations. The height of each bar indicates the proportion of the genome associated with the HBD class of the corresponding color (RK).

Here, we confirmed 428 population locations and obtained genomic data for 26 individuals by surveying all distributions of the Tibetan macaque (Fig. 1A). We conducted a comprehensive analysis combining population genomics, landscape genomics, and multi-niche modeling. This study aimed to (i) determine the effects of climatic fluctuations on the genetic landscape in Tibetan macaques by examining the population structure, demographic history, and divergence times; (ii) estimate the negative genetic effects that may persist in populations after bottlenecks by quantifying the accumulated genetic load in the population; and (iii) assessing the persistent impact of bottlenecks on the future adaptive potential of Tibetan macaques by predicting the ecological and genomic vulnerability of Tibetan macaque populations facing future climate change. Our study provides a useful guideline for understanding which populations may be at risk from climate change and can assist future conservation efforts.

## RESULTS

### Genome sequencing and variant discovery

With an average of 45.13 gigabyte per individual, we produced 1173.4 gigabyte of clean data (quality-controlled reads) (GSA: PRJCA026025) (table S1). All paired-end sequences were mapped to the reference genome assembly of the rhesus macaque (assembly Mmul_10, NCBI Annotation Release: 103, https://ncbi.nlm.nih.gov/datasets/genome/GCF_003339765.1/) (*46*), which was assembled using the highest quality reference genome of a model animal of the Macaque genus. The sequencing depth varied from 20.45× to 40.3× (mean depth: 26.50×), and the genome coverage ranged from 97.92 to 99.71% (mean coverage: 99.25%) (table S1). Among the 40,061,664 single-nucleotide polymorphisms (SNPs) identified, variants were detected in the intergenic regions and accounted for a large proportion (31.61%), whereas variants in the exons accounted for only 0.36% (table S2).

### Population structure, genetic landscape, and demographic history

To examine the population structure of Tibetan macaques in China, we first obtained larger *Fst* values between the eastern and western groups (*Fst* = 0.48792 ± 0.194108), but smaller *Fst* values within the eastern and western groups, respectively (Fig. 1B). In addition, we performed principal components analysis (PCA) and Admixture analyses. PCA revealed two nonoverlapping clusters (PCA1 = 22.07589% and PCA2 = 5.391962%), the first of which included individuals from the eastern group [including HS (representing the Huangshan Mountain of China), WYL (representing the Wuyi Mountain of China), and MS (representing the Mangshan Mountain of China)], and the second of which comprised the western group [including SC (representing the Sichuan region of China), FJS (representing the Fanjing Mountain of China), and WMS (representing the Wumeng Mountain of China)] (Fig. 1C and table S3). For *K* = 2, the lowest cross-validation score in the admixture analysis indicated two genetically separate subpopulations of Tibetan macaques, which was consistent with the PCA results (fig. S3). Further validation of the admixture algorithm and the PCA results was provided by the maximum-likelihood (ML) tree (Fig. 1D). We further uncovered the population divergence history using MCMCTree in PAML v4.9, which indicated the divergence between the eastern and western groups occurred at 1.08 million years (Ma) (Fig. 1E and fig. S4). This was coincident with population decline caused by the paleoclimatic oscillations. The results suggested that the two groups have experienced similar demographic histories. At about 0.3 Ma, the population decline led to a bottleneck, which may have been caused by a series of palaeoclimatic oscillations (e.g., the Xixiabangma Glacier and the Nayongera Glacier) (Fig. 1F and fig. S5) (*47*). Similarly, the results of stairway plot 2 showed that the eastern and western groups experienced a continuous decline in population size due to palaeoclimatic oscillations (fig. S6). This suggests that the dynamics of population size may be related to glacial cycles (*48*).

According to the analysis of genetic diversity using average genome-wide heterozygosity, we found that the heterozygosity of HS was the lowest (5.567 × 10^−4^) (Fig. 1G and fig. S7). In addition, by comparing the genetic diversity of 15 species in the genus *Macaca*, we found that the genetic diversity of the Tibetan macaques was only higher than that of the Barbary macaques (*Macaca sylvanus*), which had a conservation rank of endangered (EN) (fig. S8). At the genome level, individuals from populations that experienced recent bottleneck may have an increased probability of carrying identical-by-descent (IBD) alleles, including deleterious variants that can contribute to inbreeding depression, which may threaten the recovery process of populations (*49*). Within individual genomes, IBD alleles are generally located in extended homozygous-by-descent (HBD) segments that appear as long stretches of homozygous genotypes called runs-of-homozygosity (ROH). The expected HBD segment lengths are inversely related to the number of generations to the common ancestor and their frequency to past effective population size and individual inbreeding coefficients (*50*). Therefore, we determined the level of inbreeding within the eastern and western groups and found that both the HS and SC populations contained a higher proportion of HBD segments (Fig. 1H and figs. S9 and S10), which suggests higher levels of inbreeding. This suggests that both populations might be more vulnerable to reduced long-term viability as a result of inbreeding, which reduces their capacity to withstand climate change.

### Recent bottleneck and genetic load

**Fig. 2.**
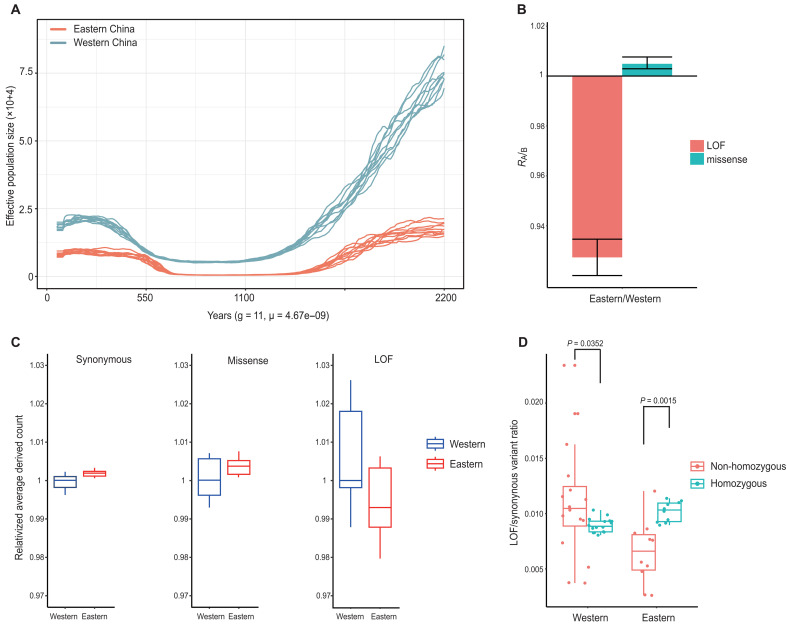
Demographic history inference and the genetic load in Tibetan macaque populations. (**A**) GONE analyses were conducted on a population-level scale, with 10 bootstrap repeats for each population, assuming a generation time of 11 years, conducted on samples sizes of *n* = 10 for eastern population and *n* = 16 for western population. (**B**) ***R****_A/B_* ratio of derived alleles for LOF and harmful missense variants. Relative number of derived alleles at LOF (red) and harmful missense (blue) sites that are frequent in one population but not in another. Error bars represent ±2 SDs. (**C**) Average number of derived alleles per annotation category. Population averages are all relative to the Western population average and shown with their error bars. (**D**) Rates of LOF variants relative to synonymous variants in homozygous and non-homozygous regions. Horizontal bars indicate population means.

GONE was used to infer the demographic history of a population within the past 200 generations from the observed spectrum of linkage disequilibrium (LD) of pairs of loci over a wide range of recombination rates in a sample of contemporary individuals (*51*). Our results showed that there were substantial bottlenecks between 1.0 and 1.2 thousand years (ka) in both the eastern and western groups; however, the effective population size (*Ne*) of the western group was consistently higher compared with that of the eastern group (Fig. 2A). To determine the effect of deleterious variation remaining in the present populations, we quantified the deleterious alleles observed in individuals of the present populations. To further investigate differences in purifying selection effects between populations, we compared the accumulated frequencies of deleterious mutations in eastern and western groups using $R_{A/B}$ analysis. Mutations classified as harmful missense (moderately deleterious) exhibited an increased frequency in the eastern group compared with the western group; however, those classified as loss of function (LOF; highly deleterious) exhibited a reduced frequency (Fig. 2B). Next, we counted the derived deleterious alleles for synonymous, harmful missense, and LOF categories, which were corrected by the count of derived synonymous alleles. The results indicated that the eastern group showed higher derived counts of synonymous and harmful missense alleles (Fig. 2C). In contrast, the eastern group exhibited lower derived counts of LOF (Fig. 2C); however, the count of homozygous derived LOF alleles was slightly increased (Fig. 2D). Together, these findings show that severely deleterious (LOF) mutations might have been reduced by purifying selection during the bottleneck, although this effect was weak.

To substantiate these findings, we performed genome evolutionary rate profile score (GERP) analysis to identify the most conserved sites across the genome (*52*), providing information on the potentially most deleterious mutations. The results of this analysis were consistent with those predicted by SNPEFF (fig. S12).

### Genomic signatures of selection and local adaptation facing climate change

**Fig. 3.**
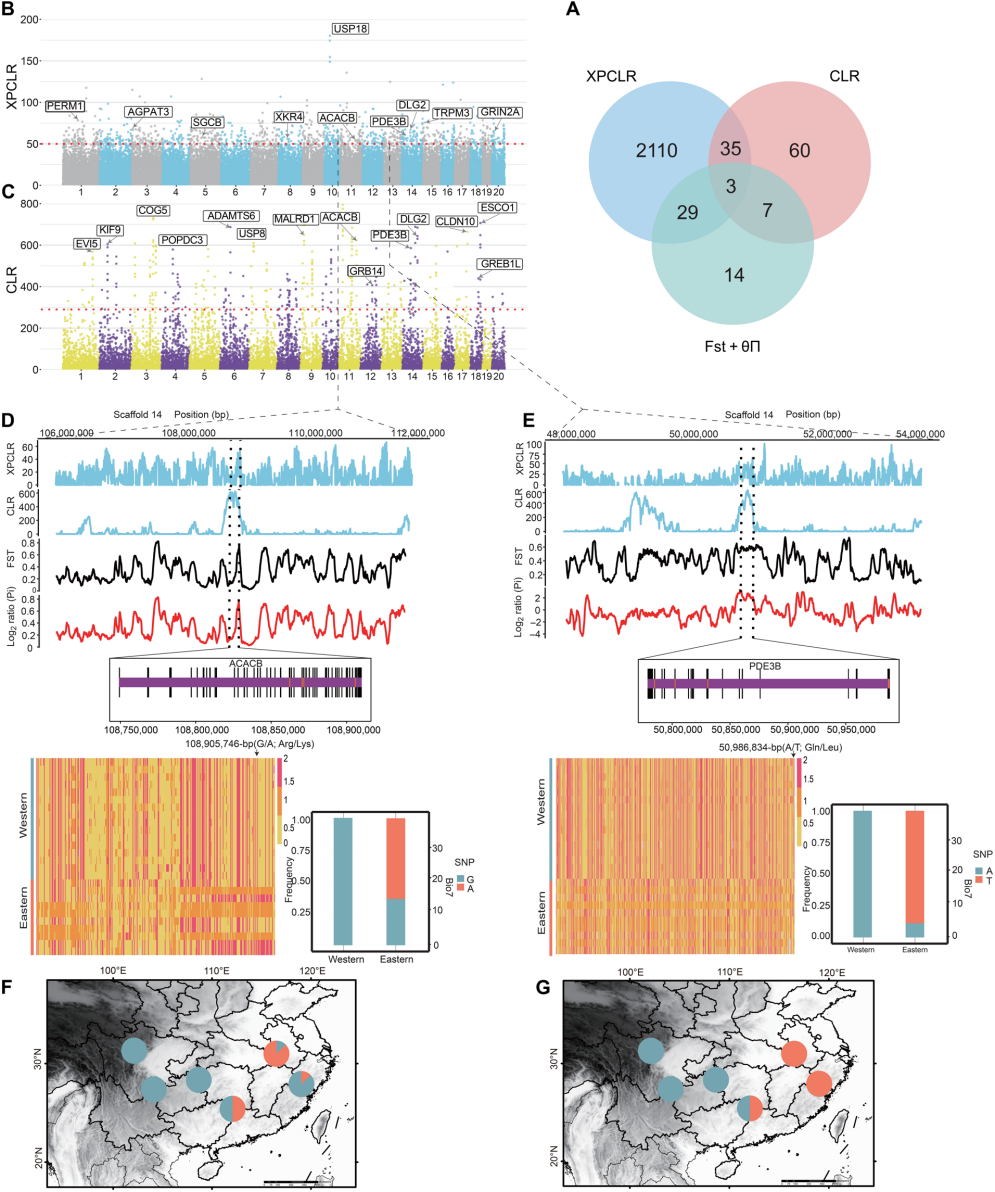
Candidate adaptive genes. (**A**) Number of candidate genes under selective sweeps identified by the three methods listed in each of the Venn diagram components. (**B**) Manhattan plot of the CLR genome scan comparing western with eastern population. The horizontal dashed line corresponds to the top 1% genome-wide significance threshold. (**C**) Manhattan plot of the XP-CLR genome scan comparing western with eastern group. The horizontal dashed line corresponds to the top 1% genome-wide significance threshold. (**D** and **E**) Signals of the gene region and genetic annotations (black bars refer to coding sequences; all missense variants are shown as orange bars). Bottom of (D) and (E) show the haplotypes of the coding region of this gene in eastern and western groups. Genotypes of SNPs, one missense SNP shows different allele frequencies in two BIO7 groups. (**F** and **G**) Allele frequencies reveal patterns of geographic change from western to eastern.

Considering the distinct geographical ranges and climatic environments of the two groups, along with the long-term genetic divergence, we primarily focused on the identification of genomic signatures of selection and local adaptation. We used an integrated approach combining the cross-population composite likelihood ratio (XP-CLR), CLR, and *Fst* + θπ ratio to scan genomic regions potentially associated with adaptation (*53*). XP-CLR is a statistical method for detecting selective sweeps based on multilocus allele frequency differentiation between two populations (*53*). CLR was able to take advantage of allele frequency differences across populations by modeling the neutral two-dimensional (2D) frequency spectrum using genome-wide data and searching for locus-specific outliers. Combining the results of the analysis of adaptation genes by three different methods, we identified three overlapping candidate genes that exhibited a signal of selection (Fig. 3A and table S5).

Functional enrichment of candidate genes (i.e., genes present in at least methods) showed positive selection for enriched pathways, such as the regulation of cyclic adenosine 3′,5′-monophosphate–mediated signaling (GO:0043949, Benjamini-Hochberg-adjusted *P* = 0.000442456), cyclic-nucleotide–mediated signaling (GO:0019935, BH-adjusted *P* = 0.002353589), and long-chain fatty-acyl–coenzyme A (CoA) metabolic process (GO:0035336, BH-adjusted *P* = 0.028792512) (fig. S14). Of these candidate genes, *ACACB* (acetyl-coA carboxylase beta), *PDE3B* (phosphodiesterase 3B), and *DLG2* (discs large MAGUNK scaffold protein 2) were strongly associated with variation in temperature annual range (BIO7), and showed high signals in all three screening methods compared with nearby gene regions (Fig. 3, B to E, and fig. S15). This suggests that they may be related to temperature local adaptation. The *ACACB* gene is involved in the regulation of fatty acid oxidation by inhibiting fatty acid and glucose oxidation and enhancing fat storage (*54*). Furthermore, we showed the distribution pattern of allele frequencies of a potential candidate adaptive nonsynonymous SNP located in the exon of *ACACB* [G/A, Arg/Lys; position: chromosome 14, 108,905,746 base pairs (bp)] (Fig. 3D). While the G allele was nearly fixed in locations with low-temperature annual range, the A allele was mostly distributed in ranges characterized by higher temperature annual range (Fig. 3D). We used Alphafold2 for 3D protein structure prediction and found that the missense mutation site is located within the carboxyl transferase domain of the protein (fig. S17, A and B). In addition, the results from another gene showed that the allele frequency of the SNPs located in the *PDE3B* coding region varied between the two groups along the gradient of the temperature annual range variable (Fig. 3E), which indicates that this gene may be a candidate for climate adaptation. PDE3B-knockout mice exhibited multiple alterations in the regulation of lipolysis, lipogenesis, and insulin secretion (*55*); thus, we selected one potential adaptive SNP located in the coding region of the *PDE3B* gene (A/T, Gln/Leu; position: chromosome 14, 50,986,834 bp) for further analysis. The results indicated that the T allele was more prevalent in populations with higher temperature annual range, whereas the A allele was more likely to be observed in regions with low-temperature annual range (Fig. 3G). The *DLG2* gene is suggested to be involved in the regulation of insulin secretion (*56*); we also selected one potential adaptive SNP located in the coding region of the *DLG2* gene (A/T, Gln/Leu; position: chromosome 14, 50,986,834 bp) for further analysis. The results suggested that the A allele was nearly fixed in locations with low-temperature annual range, and the G allele was mostly distributed in ranges characterized by higher-temperature annual range (fig. S15).

### Future ecological vulnerability and genomic offset prediction for future climate change

**Fig. 4.**
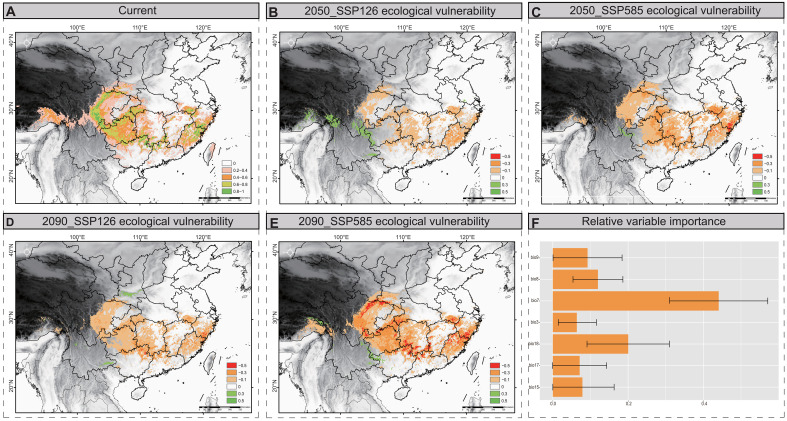
SDMs reveals current suitable habitat and future ecological vulnerability of the Tibetan macaque. (**A**) Current suitable habitat of Tibetan macaque. Green represents highly suitable habitat, yellow-green represents moderately suitable habitat, orange represents low-suitable habitat, and pink and white represent unsuitable habitat. (**B**) Spatial distribution of ecological vulnerability of Tibetan macaques under the SSP 126 climate scenario in 2050. (**C**) Spatial distribution of ecological vulnerability of Tibetan macaques under the SSP 585 climate scenario in 2050. (**D**) Spatial distribution of ecological vulnerability of Tibetan macaques under the SSP 126 climate scenario in 2090. (**E**) Spatial distribution of ecological vulnerability of Tibetan macaques under the SSP 585 climate scenario in 2090. In (B) to (E), green color represents regions with lower ecological vulnerability under different future climate scenarios, while red color represents regions with higher ecological vulnerability under different future climate scenarios. (**F**) Relative contribution of the environmental variables produced by SDMs.

When climate change surpasses what a population can tolerate in situ, the population may disperse to areas with more suitable climate conditions to mitigate the risk of extirpation (*22*). We predicted populations at increased risk for future climate change using species distribution models (SDMs), which may enable us to identify where the future suitable climate conditions are for the species. For the SDMs, we used an ensemble modeling strategy that averaged projections from five model algorithms, including support vector machine (SVM), boosted regression trees (BRT), generalized additive model (GAM), random forest (RF) and maximum entropy (MaxEnt). After removing variables that demonstrated collinearity (variance inflation factor, VIF > 10), we found that the following seven climatic variables (VIF < 10) remained: isothermality (BIO3), temperature annual range (BIO7), mean temperature of the wettest quarter (BIO8), mean temperature of the driest quarter (BIO9), seasonality of precipitation (BIO15), precipitation of the driest quarter (BIO17), and precipitation of the warmest quarter (BIO18) (table S8). With an AUC (area under the curve) ranging from 0.97 to 0.99 and the TSS (true skill statistic) surpassing 0.91 for every model, these models demonstrated a strong discriminative ability (table S9 and fig. S18). On the basis of the habitat suitability map predicted by the ensemble SDMs, the results indicated that the total area of suitable habitat currently available for this species was 1,174,495.65 km^2^ (Fig. 4A). Of the total area, the Huangshan Mountain, Wuyi Mountain, Nanling Mountain range in eastern China, and most of the western region should represent currently suitable habitats for Tibetan macaques (Fig. 4A). As to suitable habitat for this species in the future, the results indicated that its suitable habitat will be reduced by 20.4 and 30.5% in the 2050s based on shared socioeconomic pathway (SSP) SSP1-2.6 and SSP5-8.5 emission scenarios, respectively (Fig. 4, B and C). By 2090, the suitable available habitat will decrease by 42.1 and 57.2% compared with the current habitat under SSP1-2.6 and SSP5-8.5 emissions scenarios, respectively (Fig. 4, D and E). The ecological vulnerabilities of Huangshan Mountain, Wuyi Mountain, Nanling Mountain, and the northern Sichuan Province are high, which suggests that the populations within these mountains may be very vulnerable (table S10). Furthermore, the dispersal of Tibetan macaques among ranges was influenced by BIO7 (Fig. 4F), which indicates that temperature fluctuations were important for Tibetan macaque habitat selection. In addition, independent modeling of the eastern and western groups showed that the ecological vulnerability of both the eastern and western groups increased with climate change (fig. S20). The western group has a tendency to migrate northward in the future, in which the Qinling region of China may be a refuge from future climate change, while for the eastern group, because of the high degree of habitat fragmentation, it will lose almost all of its suitable habitats in the future and will face a high risk of climate change (fig. S21).

Furthermore, to determine how intraspecific variation in the genotype-climate association has driven different degrees of genomic offsets in response to climate change, we performed a GEA analysis using two approaches [latent factor mixed model (LFMM) (*57*) and redundancy analysis (RDA) (*58*)] to detect environment-associated genetic variants. Five variables with VIF <10 were selected for the RDA analyses after taking into account their ranked importance based on gradient forest analysis and the interrelations among these environmental variables. These five variables included two precipitation variables (BIO15 and BIO18) and three temperature variables (BIO7, BIO8, and BIO9). A total of 7268 and 8685 SNPs were identified by LFMM and RDA, respectively; however, only 234 SNPs were considered as candidate climate adaptation loci (fig. S22).

**Fig. 5.**
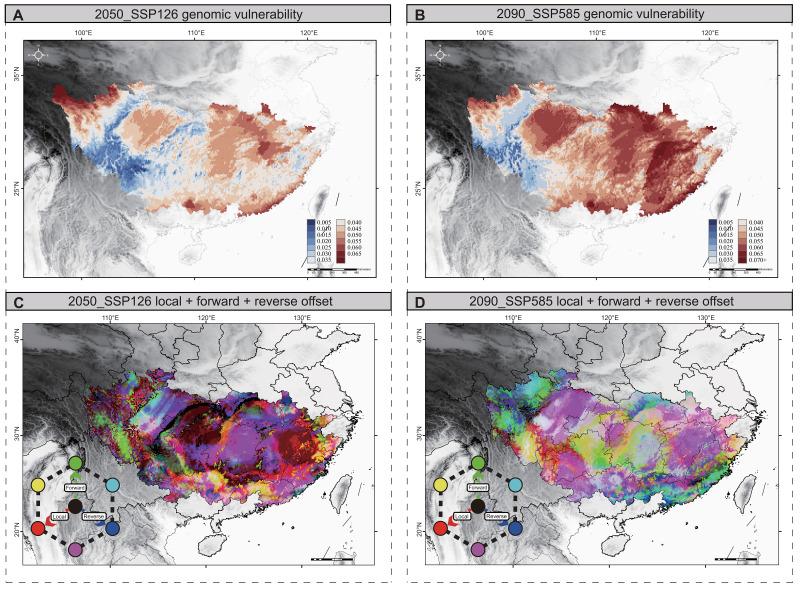
Predicted genetic offsets to future climate change under SSP 126 in 2050 and SSP 585 in 2090. (**A** and **B**) Map of the GF-predicted genetic offset under two scenarios of SSP 126 in 2050 (A) and SSP 585 in 2090 (B). The color scale from blue to red refers to increasing values of genetic offset. (**C** and **D**) RGB map of local (red), forward (green), and reverse (blue) offsets throughout the range of Tibetan macaque under SSP 126 in 2050 (C) and SSP 585 in 2090 (D). Brighter cells (close to white) have relatively high values along each of the three axes, whereas darker cells (close to black) have relatively lower values.

The analysis of the spatial pattern of maladaptation was conducted across the range of Tibetan macaques for future climate conditions by two complementary approaches. First, we focused on predicting genomic offset under future climate change by using the gradient forest (GF) approach, which is an extension of the RF approach (*30*). This method allowed for modeling the associations between the composite effects of multiple adaptive loci and different climate variables simultaneously (*59*). The results indicated that there was an obvious pattern of growing genomic offset with rising emissions when the genomic offset under several emission scenarios was compared (Fig. 5, A and B, and fig. S24). This suggests that the eastern group may be the most vulnerable to future climate change.

Furthermore, we also involved assessing the metrics of forward and reverse genomic offset using the Generalized Dissimilarity Modeling (GDM) model, which incorporated migration into the analysis alongside the classic (local) genomic offset (*24*). Forward offset can be interpreted as the relative possibility of contemporary genotype–climate associations disappearing from the landscape assuming that populations were unconstrained by migration (*24*). Reverse offset is similar to the concepts of emerging climate and emerging species assemblages (*60*), but applied to the adaptive genetic composition of populations, representing the possibility that any population in the current range will be preadapted to a particular location in the future. We found that the trends in forward genomic offset were largely similar across different maximum dispersal distances (100, 250, 500, and 1000 km); however, limiting the maximum migration lengths always resulted in a greater forward offset (fig. S26). Therefore, by determining the smallest projected offset, we could assess the forward genomic offset. Moreover, the reverse offset could be calculated by determining the smallest offset for any modern population in the present range that matches the anticipated future climate at a particular site, following the shift in focus from populations to locations (*24*). Although the predicted patterns of local, forward, and reverse offsets varied throughout the range of Tibetan macaques, the HS population in the eastern was consistently predicted to have relatively high local, forward, and reverse offsets (Fig. 5, C and D, and fig. S27), indicating that the HS population was less resilient to future climate change.

## DISCUSSION

Climate change causes increasingly severe and extreme weather changes, which results in decreased survival for wildlife (*61*). The ecological and genetic adaptation of wildlife to climate changes poses a question in evolutionary and conservation biology (*62*). As a near-threatened primate endemic to China, assessing its response and vulnerabilities to climate change is imperative to understanding how this species will cope with climate change in the future. Analyses were conducted by combining population structure, demographic history, recent bottleneck, genetic load, local adaption, range shift, and genomic offset. We examined the effect of climate fluctuations on the ecological and genomic vulnerabilities as well as the adaptation potential under future climate change. Our results indicate that climatic fluctuations might play an important role in shaping demographic trajectories and genetic structure, and caused severe bottlenecks in the recent past. We also found that the HS population faces a higher risk of climate change because of ecological and genomic vulnerability. Furthermore, we suggest that BIO7 might be one of the important factors, which may affect suitability of habitat for the Tibetan macaques.

Climate fluctuations are important drivers of biodiversification across a range of taxa (*63*). The Pairwise Sequentially Markovian Coalescent (PSMC) results indicated that climate change during the Xixiabangma Glaciation resulted in a dramatic decline in *Ne* across all populations and divided the Tibetan macaque populations into two genetically homogeneous groups in eastern and western China (MCMCTree: 1.08 ± 0.33 Ma), which did not support the previous view of a division into four subspecies (Fig. 1, D and F) (*50*). Because of the long-term isolation between populations, we suggest that the eastern and western groups represent two distinct evolutionarily units (*64*). Moreover, the GONE results indicated that the *Ne* of the eastern and western groups simultaneously declined sharply approximately 1.1 ka, which indicates that there was an extremely dramatic demographic bottleneck (Fig. 2A), coinciding with a period of climatic fluctuation. During bottlenecks, rapid fixation of polymorphic loci by drift drastically reduces the genetic diversity of the population. Also, genome-wide diversity is an important predictor of population fitness and adaptive potential, which is the ability to respond to future environmental change (*65*). We hypothesize that recent severe bottlenecks drastically reduced the potential adaptive capacity of the Tibetan macaque to future climate change and contributed to the negative genetic effects emerging and persisting during its population decline (*66*).

Since bottlenecks can lead to a rapid loss of genetic variation and reduce the adaptive potential of a population (*67*), we estimated the negative genetic effects that may persist in the population after the bottleneck. Our results support the expected outcomes based on previous theoretical expectations (*51*, *65*, *68*) and empirical observations in other taxa (*50*, *51*, *53*). Specifically, we observed a distinct pattern of highly deleterious and moderately deleterious variation after the bottleneck (Fig. 2B). Theory predicts that population bottlenecks reduce the proportion of segregating sites with deleterious mutations but also increase the frequency of deleterious variants at some loci that survive the bottleneck (*65*), which increases the proportion of homozygosity leading to increases of the realized load and inbreeding depression (*51*, *68*). In our study, we also found that there was 0.37% higher homozygosity compared with heterozygosity in the eastern group (Fig. 2D), which indicates that our results are consistent with the theoretical prediction. Furthermore, we compared the number of absolute derived alleles between the eastern and western groups. We found that derived alleles of moderately deleterious variants (i.e., harmful missense mutations) were increased in frequency (Fig. 2C). Although there was only a slight increase, even small differences in genetic load could have an impact during evolution. We hypothesize that the eastern group may be affected primarily by genetic drift with purifying selection (*65*, *69*). Although purifying selection could decrease the frequency of highly deleterious mutation sites, such as LOF, a portion of moderately deleterious mutation sites randomly drift to high frequencies, rendering purifying selection ineffective (*70*). Therefore, we suggest that random fixation of moderately deleterious mutations causes long-lasting damage to adaptation to future climate change, although the *Ne* of the Tibetan macaques has recovered over the past 20 generations (Fig. 2A).

However, genomic erosion is often only noticeable many generations after the onset of the immediate threats that lead to population decline (*65*, *69*). Thus, there is a time lag between the demographic and genomic impacts of the threats. Most deleterious mutations are initially rare, and, hence, it can take many generations of drift for them to increase in frequency, in a process referred to as “genetic drift debt” (*71*–*73*). Only once these mutations are common enough do they become homozygous and reduce the mean population fitness. The fitness effects of recessive deleterious mutations that were initially masked as heterozygotes then become expressed as homozygotes. The masked load is thus converted into a realized load (*74*, *75*), changing the constitution of the genetic load. Inbreeding can accelerate this process, resulting in inbreeding depression. We observed higher realized load and lower masked load in the eastern group compared to the western group, implying an accumulation of deleterious alleles in homozygous state (fig. S12). Similar patterns were also found in the pink pigeon (*76*, *77*), the whooping crane (*74*), the Scandinavian wolf population (*71*, *78*), the Florida panther (*79*), and the Scandinavian Arctic fox population (*80*), all of which represent populations that have suffered from severe bottlenecks followed by inbreeding in the relatively recent past. Our study therefore emphasizes the use of genomic tools to provide a comprehensive framework for determining inbreeding depression, genetic load, and adaptive potential, and to develop rational conservation strategies to enhance species survival.

The minimum sample size for population genetics and conservation studies has been a hotly debated topic (*81*). For most population genetic studies, an adequate sample size would be the one that allows for reliable estimation and comparison of genetic diversity and genetic subdivision parameters among populations. However, for protected species that are difficult to sample, estimating genetic diversity parameters in large natural populations using limited sample sizes is one of the central issues in population and conservation genetics research. Fumagalli (*82*) showed that the accuracy of inferred genetic diversity increases with sample size despite low sequencing depth. Not only that, for GONE, which relies on LD to compute the nearest *Ne*, the accuracy of population parameter estimates cannot be compensated by more SNPs if the sample size is small. In addition, because of sample size limitations, it may not be possible to accurately detect all deleterious mutations, leading to a possible underestimation of genetic load. As noted by Kardos (*83*), very large sample sizes may be required to obtain sufficient statistical power when assessing genetic load. Despite these limitations, our study provides a preliminary understanding of the genomics of Tibetan macaque populations and provides basic data for identifying conservation priorities.

Studies indicate that if specific genetic variations facilitate adaptation to extreme environment, their frequencies should shift along relevant environmental gradients (*84*). Our results indicate allele frequency patterns of SNPs in the *PDE3B* and *ACACB* coding regions along the BIO7 gradient (Fig. 3, F and G). Furthermore, LD was observed in all groups and there are many factors that can contribute to LD. In addition, we identified additional genes that may play an important role in climate adaptation, such as genes related to muscle production and differentiation, including *USP18*, *MYPN*, and *SGCB* (Fig. 3, B and C). Studies have shown that the muscle plays an important role in maintaining thermal homeostasis (*85*). These genes may enhance the cold tolerance of this species. Also, transient receptor potential melastatin 3 (*TRPM3*) was identified by both XP-CLR and CLR (Fig. 3, B and C), which indicates that they may play a key role in heat stress tolerance and thermal sensitization. However, this might be part of a polygenic network involved in local adaptation. There is a large body of recent evidence for polygenic climate adaptation in many species (*86*, *87*). This is consistent with the expectation of genetic complexity for many climate-related traits, with differences between populations arising from the small contributions of many segregating loci (*88*). Polygenic traits are likely to be rapidly responsive to environmental change because the time to reach an adaptive optimum is inversely proportional to the number of loci controlling that trait (*89*).

Local climate adaptation is likely to result in differences between populations with extirpation risks following sustained climate change (*90*). Thus, assessing the ecological and genomic vulnerability of different populations is extremely important for planning and prioritizing conservation strategies. In this study, we projected population-specific genomic offset for future climate change based on the intraspecific variation of the species. Genomic offset is a measure of the amount of genetic change needed to adjust to climate conditions. The populations with the greatest genomic offset are those that have to adjust the most. In response to future climate change, we found that the eastern group, particularly the HS population, exhibited larger genomic offsets (Fig. 5, C and D), which suggests that the risk of nonadaptation will increase in the future. Moreover, ecological vulnerability analysis revealed that human efforts to adopt climate protection measures would have a positive impact on the conservation of the Tibetan macaque habitat, as large areas of suitable habitat were severely reduced under the SSP5-8.5 emission scenarios. However, there was less suitable habitat decline in the SSP1-26 emission scenario (Fig. 4). Studies indicate that climate change and human activities have effects on primate viability and distribution over time. We thus suggest that rapid urbanization and increasing population density in eastern China may also be an important reason for the increased ecological vulnerability of Tibetan macaques. In conclusion, our results suggest that the eastern group, particularly the HS population, may face higher risk of climate change in the future duo to their higher levels of inbreeding, lower genetic diversity, and higher ecological and genomic vulnerability, whereas the western group will need to cope with the risk of habitat degradation under future climate change, despite experiencing a relatively minor genomic offset.

Through the integration of multidisciplinary approaches, we enhanced our appreciation of climate change-driven vulnerability, which has provided us with a better understanding of how post-bottleneck species will react to future climate change. This may be used to implement conservation measures and to reduce the negative effects of climate change. According to the latest IUCN Red List classification, Tibetan macaques are currently nearly threatened, and we suggest that this likely underestimates the current level of threat to the Tibetan macaque. This is because either locally or elsewhere in its range, no populations have preadapted to the environment that will exist in this area in the future. Therefore, we recommend raising the protection level of Tibetan macaques. In addition, considering that the eastern group, especially the HS population, are also unable to mitigate climate change through migration or dispersal to more suitable habitats, we suggest that ecological corridors be constructed in the future in the Huangshan Mountain, Mangshan Mountain, and Wuyishan National Parks to ensure the connectivity of the eastern group’s habitats and to better use the effectiveness of the national parks in protecting the species. Furthermore, we also suggest that individuals may need to be introduced into the HS population for genetic rescue, but we caution that such gene flow may also introduce other deleterious alleles, so long-term attention to this population is still needed in the future.

## MATERIALS AND METHODS

### Sampling, genomic data generation, and processing

#### *Sample collection*, *DNA extraction*, *and sequencing*

We collected tissue and/or blood samples from 26 Tibetan macaques in the field and other 2 individuals’ genome data from Tan’s study (table S1) (*91*). Using the DNeasy Blood and Tissue Kit (Qiagen, Hilden, Germany) and the manufacturer’s instructions, DNA was obtained from each sample. For the purpose of genome sequencing, a library was built using around 3.3 μg of genomic DNA from each sample, using the Watchmaker DNA library prep kits with fragmentation (Watchmaker Genomics, USA). Following the manufacturer’s instructions (Illumina Inc., San Diego, CA, USA), sequencing libraries were built (insert size: 350 bp) and were sequenced at the paired-end 150 bp on the llumina NovaSeq 6000 PE 150 platform in Anoroad, Beijing, China. The average median depth of the samples’ sequencing was 26.50× (20.45 to 40.3×).

#### Ethics statement

Sample collection was carried out in accordance with the approved guidelines of the Good Experimental Practices adopted by the Institute of Zoology, Chinese Academy of Sciences (CAS). All experimental procedures and sample collections were conducted under the supervision of the Committee for Animal Experiments of the Institute of Zoology, CAS.

#### Data filter and variant detection

Using the default settings of Trim Galore v0.4.4, all FASTQ data adapter material was trimmed, and 5 bp were eliminated from each read’s 5′ and 3′ ends (https://www.bioinformatics.babraham.ac.uk/projects/trim_galore/). FastQC v0.11.9 (https://www.bioinformatics.babraham.ac.uk/projects/fastqc/) was used for quality control (*92*). BWA-MEM v0.7.17 (*93*) was used to map the reads to the reference genome of the rhesus macaque [assembly Mmul_10 (*46*), NCBI Annotation Release: 103], using the default parameters. Following mapping, GATK IndelRealingner was used to realign readings around indels (*94*). Duplicates were eliminated using the Picard Mark Duplicates tool (https://sourceforge.net/projects/picard) after sorting using Samtools v1.7 (*95*). In addition, for repetitive regions and low-complexity DNA sequences, we used RepeatMasker for marking and removing (*96*). Next, SNPs were found using the collaborative genotyping strategy’s GATK best practice process. Using the Haplotypecaller module and the command “-genotyping-mode DISCOVERY –min-base-quality-score 20 -stand-call-conf 30 –emit-ref-confidence GVCF”, we used the GenotypeGVCFs module to combine all of the GVCFs to carry out joint genotyping. Following that, the parameters depth (QD) < 2.0, root mean square of mapping quality (MQ) < 40.0, Fisher Strand (FS) > 60.0, HaplotypeScore >13.0, and MQRankSum ≤12.5 were used to filter the raw variant calls. In addition, using VCFtools v0.1.15 (*97*), SNPs with missing rates ≥0.1, the quality <20 (phred-scale), and non-biallelic SNPs were filtered out. Last, 40,061,664 SNPs remained for further analysis.

### Population structure and characteristics

#### Inference of kinship in population

The small population size and population structure can result in the sampling of closely related individuals, which would inflate the estimated level of inbreeding. To avoid this, we evaluated the relatedness of all samples in each population using the default settings of KING v2.1.3 (*98*) to exclude closely related individuals within the population. Closely connected individuals were those in pairs (twins and first degree relationships) with a kinship coefficient > 0.177. The Rscript from the KING program was used to generate the pairs individual kinship diagram (http://people.virginia.edu/~wc9c/KING/hapmapkin0.R) (*98*).

#### Population structure and phylogenetic analysis

One white-cheeked macaque (*Macaca leucogenys*) and one Taiwanese macaque (*Macaca cyclopis*) served as outgroups in the phylogenetic analysis. An ML tree was built for the autosomal whole-genome SNP set [minor allele frequency (MAF) ≥ 0.05] using TreeBeST v1.9.2 (http://treesoft.sourceforge.net/treebest.shtml), to examine population structure and phylogenetic analysis. EIGENSOFT v6.1 was used to conduct the PCA. To ascertain the eigenvectors’ level, a Tracy-Wildon test was applied. We examined the ancestral populations using Admixture v1.3, running the analysis 20 times for each K and using coancestry clusters ranging from *K* = 1 to 7. The probability of each run was used to determine which was the best run. Using a cross-validation process, the Admixture program determined the optimal value of the coancestry cluster (K).

#### Genetic diversity and characterization of inbreeding

ANGSD v0.938 was used to assess the individual degree of heterozygosity for each sample; low-quality reads and bases were eliminated using the parameter “-minQ 20 -minmapq 30”. Using VCFtools v0.1.15, nucleotide diversity (π) was determined by a sliding window method with windows of 50 kb and a step of 25 kb (*97*). The R package ZooRoH v0.3 was used to define individual inbreeding via model-based methos by hidden Markov models based on the genome-wide SNP collection (MAF ≥ 0.05). This package assesses the contribution of levels of inbreeding from different generation and classifies HBD segments into generation-based classes. The expected length of the HBD segments is 1/*Rk* Morgans, where *Rk* is the exponential distribution rate for each class (*k* is the class number). To obtain more classes for long HBD segments, we considered a power of 2 for the coefficients of *Rk*, which were set from 2 to 512 (1, 2, 4, 8, 16, 32, 64, 128, 256, and 512).

#### Demographic history

We analyzed demographic history using two methods based on the sequentially Markovian coalescent, PSMC v0.6.5-r67 (*99*) and based on site frequency spectra (SFS), stairway plot2. PSMC has the strengths in resolving long-term demographic history but has limited power for resolving history during the past few thousand generations. In contrast, stairway plot 2 has better resolution of demographic history toward the recent past, but depending on overall genetic variation, it shows poorer resolution into the more distant past. PSMC was run with default parameter settings, namely time intervals (-p) were set to “4 + 25 * 2 + 4 + 6” with maximum of 25 iterations (-N). Although these settings were optimized for humans (*99*), splitting of the first time window (-p “2 + 2 + 25 * 2 + 4 + 6” and -p “1 + 1 + 1 + 1 + 25 * 2 + 4 + 6”) produced unrealistically high *Ne* estimates at the most recent time interval-a typical sign of model overfitting. We therefore set different parameters for PSMC to avoid unrealistically high *Ne*. The past million years (Ma)’ worth of atmospheric surface air temperature data were obtained from NCDC (http://ncdc.noaa.gov/). For the Tibetan macaque, PSMC plots were scaled using a mutation rate (μ) of 4.67 × 10^−9^ and a generation time (g) of 11 years (*100*). Using MEGA v7 to extract fourfold degenerate (4D) sites, they were concatenated using MCMCTree in PAML v4.9 to estimate divergence times. We placed limits on two nodes (*M. cyclopis*, which is 3.3 to 4.3 Ma, and *M. leucogenys*, which is 1.8 to 2.4 Ma) to calibrate the molecular clock (*91*). To verify whether the stationary distribution was convergent, the analysis was performed twice, and the outcomes from each run were contrasted. In addition, given that the largest differences exist between eastern and western, and the fact that for GONE, if the sample size is very small, the accuracy of population parameter estimates cannot be compensated for by a larger number of SNPs. Therefore, we used GONE to estimated recent effective population size (*Ne*) for the eastern and western groups (*51*). GONE calculates LD between pairs of SNPs over a range of recombination rates and finds the series of *Ne* that best explains the observed LD spectrum (*51*). We ran all simulations for 2000 generations calculated in 400 bins but only present results for the most recent 200 generations. To account for variation in *Ne* estimates across runs, we replicated every experiment 10 times using a fresh subsample of 300,000 SNPs (similar to bootstrapping).

### Genetic load analysis

We estimated the genetic load in Tibetan macaques’ genomes using two approaches. First, SNPEFF v4.3 was used to annotate SNPs (*101*). Before this, to infer the ancestral state of each site, we converted the references for the white-cheeked macaque and Taiwanese macaque into FASTQ reads by sliding across the genome in nonoverlapping windows of 100 bp and transforming each window into a separate FASTQ read. The resulting FASTQ reads were then mapped to the rhesus macaque reference genome with bwa mem v0.7.17, slightly lowering the mismatch penalty (- B 3) and removing reads that mapped to multiple regions. Mapped reads were realigned around indels using GATK (https://gatk.broadinstitute.org/hc/en-us) IndelRealigner. Next, we converted the mapped reads into a haploid FASTA consensus sequence, excluding all sites with depth above one (as such sites contain at least one mismapped read) using ANGSD (http://popgen.dk/angsd/index.php/ANGSD) -dofasta. The ancestral allele at a locus was then determined as the majority allele found in genomic alignment of the white-cheeked macaque and Taiwanese macaque. Sites where the ancestral allele could not be identified were excluded (*52*).

Then, three categories were classified from the annotated results: (i) low-impact variants that are likely to be not deleterious (i.e., synonymous), (ii) moderate-impact variants that are likely to modify the protein effectiveness (i.e., missense), and (iii) high-impact variants are likely to disrupt the protein function (i.e., LOF) (*101*). Furthermore, the measure we used to diagnose the deleteriousness of missense mutations is the Grantham score (*102*), a measure of physical/chemical properties of amino acid changes. The score ranges from 5 to 215, with scores bigger than 150 designated as radical or deleterious. We further categorized missense mutations into deleterious and benign as follows: (i) deleterious: missense mutations with Grantham scores equal to or more than 150; (ii) benign: missense mutations with Grantham scores less than 150.

We first determined the genetic load using the recently developed method known as the ***R****x/y* ratio to compare patterns of detrimental burden at the population level. Two groups of Tibetan macaques were identified: the eastern group and the western group. To compare the two groups, we first determined the observed derived allele frequency in group A using the formula $f_{i}^{A}=d_{i}^{A}/n_{i}^{A}$ at each site, where $n_{i}^{A}$  is the total number of alleles and $d_{i}^{A}$ is the number of derived alleles detected in population A. In population B, parameter $f_{i}^{B}$ was defined in a similar way. To calculate $L_{A,B}\left(C\right)$ , the following formula was used

$$L_{A,B}\left(C\right)=\frac{\sum_{i\in C}f_{i}^{A}\left(1-f_{i}^{B}\right)}{\sum_{i\in I}f_{j}^{A}\left(1-f_{j}^{B}\right)}$$

where *I* is a group of intergenic sites and *C* is a specific class of protein-coding sites. The proportion of derived alleles present in population *A* relative to population *B* was then calculated as follows

$$R_{A/B}\left(C\right)=L_{A,B}\left(C\right)/L_{B,A}\left(C\right)$$

To estimate the variance of $R_{A/B}\left(C\right)$ , we used a 100-block jackknife at the locations of the set. We also counted the total number of derived alleles per site per individual and the count of those in homozygous state. We corrected these derived allelic counts by dividing them by the total count of derived synonymous site (i.e., low-impact variants), following (*103*). For each allele count comparison (across all iterations), we tested whether the difference between eastern and western groups was significant with the function *t* test in R.

Second, we measured mutations at sites under strict evolutionary constraints that may have essential functional roles. We calculated the GERP for each locus in the Tibetan macaque genome using the GERP++ software (*104*). Mutations occurring at highly conserved loci (i.e., higher GERP scores) may be more deleterious. The individual masked load was calculated as the sum of the GERP scores of all deleterious derived alleles in heterozygous genotypes, divided by the number of called genotypes per individual. The realized load was calculated as the sum of GERP scores of deleterious derived alleles in homozygous genotypes divided by all called sites in the genome. On the basis of the distribution of synonymous and deleterious nonsynonymous mutations, we set a GERP score threshold of 4 to define a mutation as potentially deleterious (*105*).

### Selection signature analysis

The genomic area was screened using a combination of the XP-CLR, CLR and *Fst* + θπ ratio in selective sweeps. We categorized each individual into high and low groups based on the mean annual temperature value of the site where it was located, where eight individuals were selected for the high mean annual temperature group, five individuals from the HS population, and three individuals from the WYL population, and eight individuals were selected for the low mean annual temperature group, six individuals from the SC population, and two individuals from the WMS population (table S7). Between the eastern and western groups, the XP-CLR approach developed by Chen (*53*) is computed. The XP-CLR scores were constructed by down-weighted the contributions of strongly correlated variations (*r*^2^ > 0.95), and the grid points spread by 5000 bp were used to calculate the scores, with a maximum of 1000 SNPs in a 50-kb window. The CLR test was included in SweepFinder2, which directly takes into account the nonequilibrium SFS and does not rely on a comparison method. For each group, we used the folded SFS to prevent biases in the reference of the ancestral allelic states. We used the empirical frequency spectrum derived from all SNPs to estimate the CLR for every 5000-bp grid interval throughout the genome. Using the VCFtools with 50-kb genomic bins and a 25-kb step, population differentiation (*Fst*) between the western and eastern group was determined. For the same bins, pairwise nucleotide diversity (θπ) was calculated. Less than one bin length scaffolds were not included. Using the R package clusterProfiler v3.16, we performed KEGG pathways analyses and GO enrichment analyses for putative genes on the datasets of overlap 74 genes that were chosen by at least two techniques. The 3D structure of proteins was generated by AlphaFold2, and the best model determined by the pLDDT score was selected. The 3D structure was visualized by PyMOL (https://pymol.org/2/).

### Ecological niche modeling and ecological vulnerability

Using the R package “sdm”, we performed an ensemble modeling strategy to model niche suitability in both present and future climates. If climate change is severe in the future, species may risk leaving their current climates. However, since most primates are found in tropical and subtropical regions (*192*), in addition to the fact that Tibetan macaques are endemic to China, we selected China and Central and Southeast Asia as the study area for modeling. According to Allouche *et al.* (*106*), models were only approved if they had respectable TSS and AUC values (TSS > 0.8, AUC > 0.9). On the basis of the weighted average of five independent models: RF, MaxEnt, GAM, BRT, and SVM, we created an SDM ensemble model. To reduce spatial autocorrelation, 428 location records that matched the resolution of the environmental data and fell within a geographical distance criterion of 5 arc-min (about 9.3 km) were used to build the models. The vifstep function in the R package “usdm” was used to solve collinearity concerns for 19 bioclim variables from the WorldClim (https://worldclim.org/). The VIF for each variable is determined using the vifstep function, which then successively eliminates the variables with the greatest VIF until no variables with a VIF larger than 10 are left. To create a presence-absence matrix of species, a total of 10,000 background sites were randomly produced within a land study region covering 108° to 121°E and 20° to 35°N, which encompassed the known ranges of Tibetan macaque. Using 10 iterations of the subsampling and bootstrapping replication techniques, the occurrence data were divided into calibration (70% of the data) and validation (30% of the data) datasets to assess each model. Using a single global circulation model (BCC-CSM2-MR) and two SSPs (SSP1-2.6 and SSP5-8.5), models were projected to the future (from 2041 to 2060, and from 2081 to 2100) to estimate the distribution of Tibetan macaque. However, a major limitation of traditional modeling approaches is the neglect of intraspecific climatic adaptations and differences in population responses to climate change, which can lead to biased predictions and conservation efforts, highlighting the importance of incorporating intraspecific climatic adaptive variation into modeling (*107*, *108*). In this study, the eastern and western groups were more different and may live under different climatic conditions, so independent modeling of the eastern and western groups could improve the specificity of predictions. On this basis, we further modeled the eastern and western groups independently. Furthermore, we calculated ecological vulnerability *V*_E_ as the difference between future ( $S_{\text{futur}e}$ ) and present ( $S_{\text{present}}$ ) climatic suitability values (*198*)

$$V_{E}=S_{\text{future}}–S_{\text{present}}$$

### Identify SNPs associated with climatic variables

To account for the false positives that are frequently observed in individual genotype-environment programs, we used two methods, LFMM and RDA, to find SNPs that have a strong correlation with the top climatic variables found by gradientForest. We conducted five different MCMC runs with a latent factor of K = 2 for the LFMM*. P* values from each of the five runs were added together, and false discovery rate correction with *P* < 0.05 was applied to account for multiple testing. The R package vegan v2.5-7 (https://CRAN.R-project.org/package=vegan) was used to perform RDA analysis. To construct the loading value (or “species scores” in vegan terminology), only the RDA axes with a significance level of *P* < 0.05 were kept. If the loading values of an SNP were more than two SDs from the average loading values, the SNP was considered to be an outlier. SNPs found using both techniques were considered to be climate-associated SNPs (*22*).

### Genomic offset assessment

For each sampling location, we downloaded future (from 2041 to 2060 and from 2081 to 2100) environment data for the 19 bioclim variables from the WorldClim CMIP6 dataset of a single global circulation model (BCC-CSM2-MR). Each of the two future environmental datasets consists of two SSPs: SSP1-2.6 and SSP 5-8.5. To assess the genomic offset to future climate change, we used three distinct methodologies.

Firstly, we GF analysis in conjunction with “gradientForest” in R to determine genomic offset for Tibetan macaque throughout their range (*26*). GF is a machine-learning regression tree-based method developed to study allele frequency turnover in genomic datasets after it was first used to identify species turnover in community ecology datasets. To estimate the genomic offset under the various future scenarios, we constructed a GF model. ArcGIS 10.2 was used to map the genomic offset and show its regional distribution. The genomic offset was computed as a metric for the Euclidean distance of the genomic composition between the present and future anticipated climates.

Secondly, we also evaluated genomic offset using the same process as GF modeling by using the distance-based approach GDM. Pairwise *Fst* matrices across populations were rescaled between 0 and 1 after being calibrated for climate-associated SNPs using diversity. We used gdm.transform to predict genotypic climate association across the range of Tibetan macaque.

Using the two genomic offset metrics of local, forward, and reverse offset, we predicted the geographical locations under future climatic conditions where the genotype-climate link will be most affected. To be more precise, the local offset was computed by assuming no dispersion or gene flow and estimating genetic differentiation for climate-associated SNPs between present and future climates at the same location (that is, multidimensional Euclidean distance for GF and *Fst* for GDM) (*109*). Under the assumption that populations have infinite potential for dispersal, forward offset represents the anticipated disturbance in the genotype-climate relationship. Genetic differentiation between all grids from the future climate and every grid from the current climate is estimated using the fitted GF and GDM results. The forward offset was defined as the minimal expected Euclidean distance/*Fst* from the pool of distance values across all futures. Elevated forward offset values suggest that the current genotype-climate correlations within the residing populations may vanish from the environment (*109*). We also examined the forward offset’s sensitivity to dispersal restrictions and examined how forward offset changed when the maximum permitted migration was restricted to distinct distance classes, including 100, 250, 500, and 1000 km.

Reserve offset is computed from the current climate to the future climate, whereas forward offset is derived from the current climate. Genetic divergence between all grids from the current climate and every grid from the future climate was projected. The reverse offset was defined as the lowest anticipated offset from the pool of offset predictions across all current grids. A measure of how the genotype-climate relationship is expected to be in the future at a particular site in comparison to the genotype-climate association currently present at any place across the range under the current climate is provided by reverse offset. Moreover, as shown in (*24*), we mapped these three measurements to the red, green, and blue bands of an RGB picture, respectively, to see local, forward, and reverse offsets concurrently.
