# Supplementary material for: Integrating population genomics and environmental data to predict adaptation to climate change in post-bottleneck Tibetan macaques
Source: Sci Adv. 2025 Jul 9;11(28):eadw0562. doi: 10.1126/sciadv.adw0562 (PMC12239957; doi:10.1126/sciadv.adw0562)
Supplement: Supplementary file 1 — Figs. S1 to S27 Tables S1 to S13 [file sciadv.adw0562_sm.pdf]

Science Advances
AAAS

Supplementary Materials for

Integrating population genomics and environmental data to predict adaptation to climate change in post-bottleneck Tibetan macaques

Yang Teng *et al.*

Corresponding author: Ming Li, lim@ioz.ac.cn; Jiwei Qi, qijiwei@ioz.ac.cn; Jing Li, ljtjf@126.com; Jinhua Li, jhli@hfnu.edu.cn

Sci. Adv. **11**, eadw0562 (2025)
DOI: 10.1126/sciadv.adw0562

This PDF file includes:

Figs. S1 to S27
Tables S1 to S13

Supplementary Figures

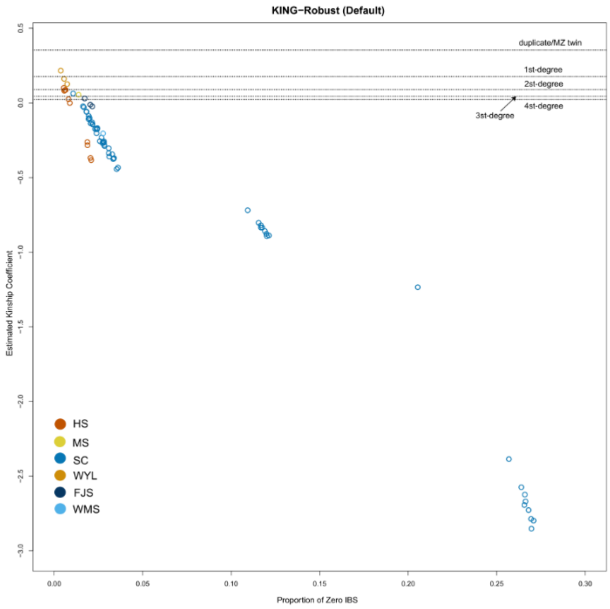

Supplementary Figure 1. Individuals' kinship within Tibetan macaque populations. Zero IBS indicates nonIdenticalByState. The dashed lines show the kinship thresholds (duplicate/MZ twin, 1stdegree, 2nddegree, 3rddegree).

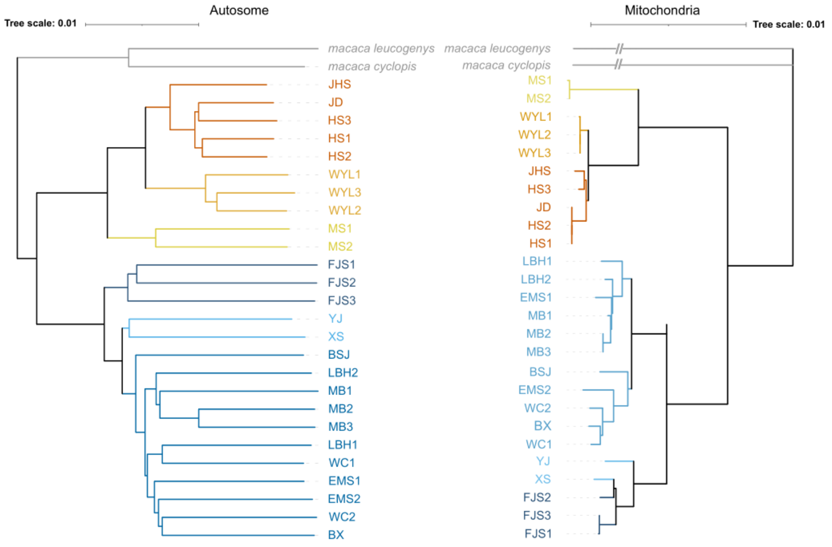

Supplementary Figure 2. Phylogenetic analysis of Tibetan macaque autosome SNP and mtDNA from all individuals based on NJ method. The label colors represent sampling populations.

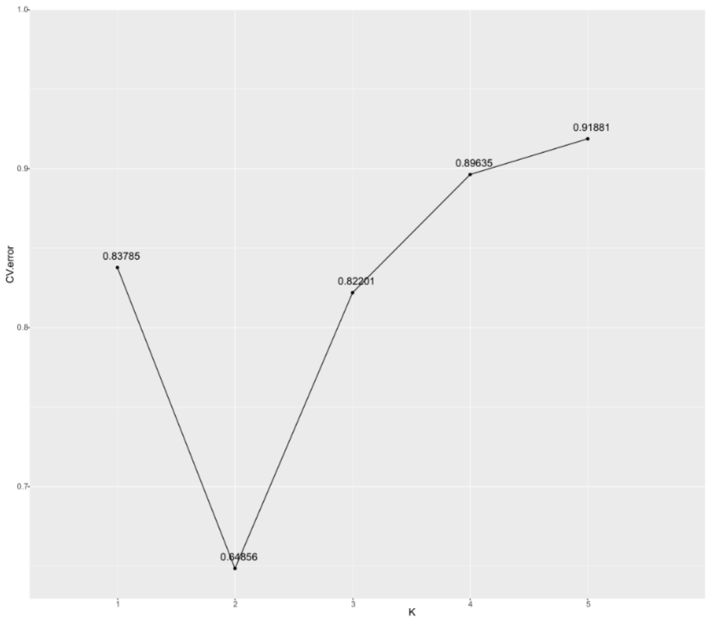

Supplementary Figure 3 Crossvalidation (CV) error for varying values of K in the Admixture analysis. Minimum of estimated CV error on K = 2 suggests the most suitable number of ancestral populations.

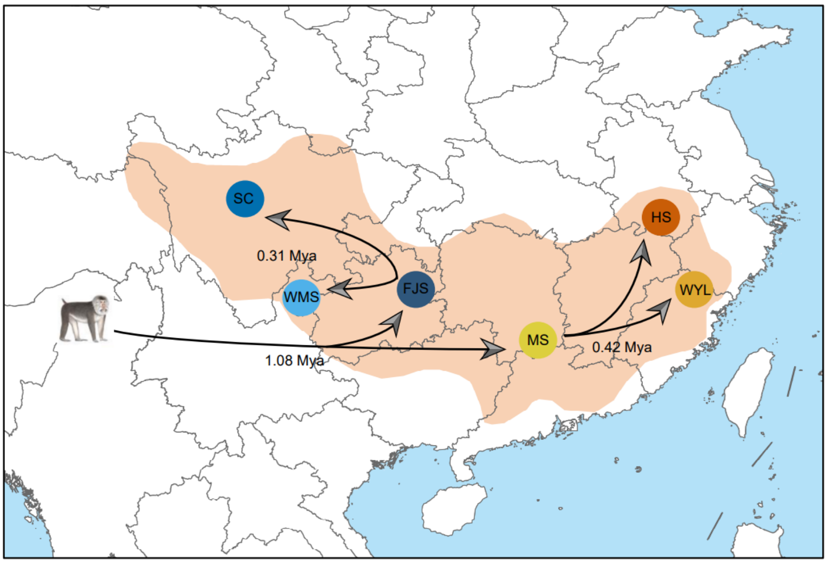

Supplementary Figure 4. Scenario for the radiation of Tibetan macaques.

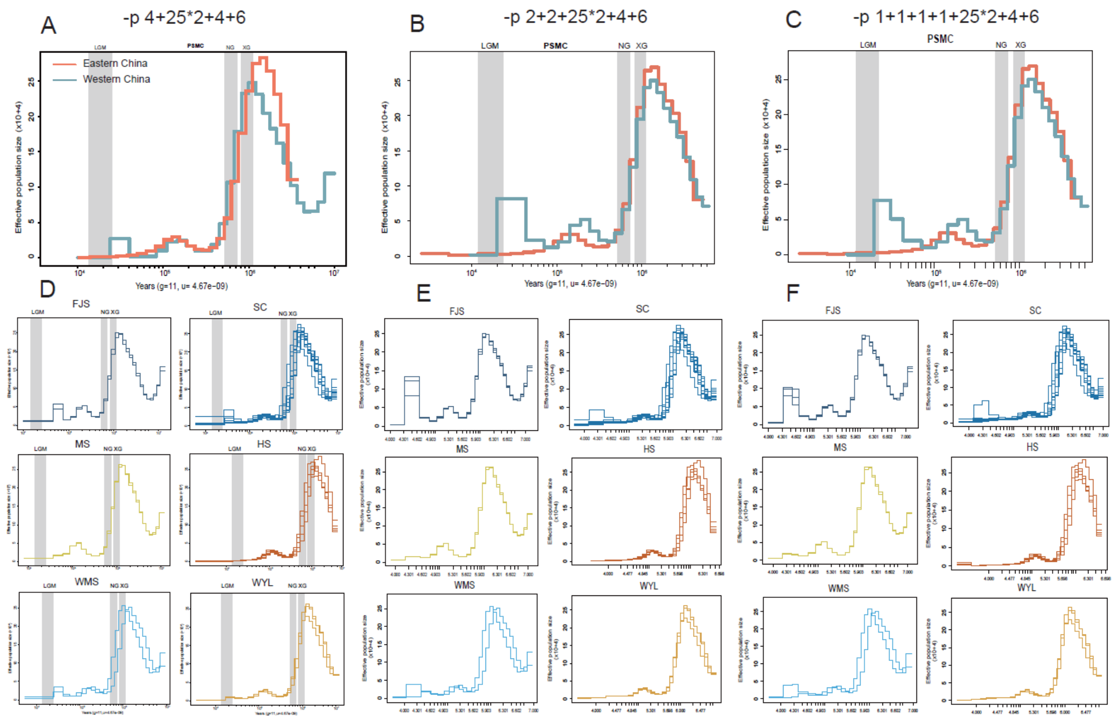

Supplementary Figure 5. Demographic history of 6 Tibetan macaque populations under different parameters of PSMC. (A) Historical dynamics of eastern and western groups under p 4+25*2+4+6 parameter. (B) Historical dynamics of eastern and western groups under p 2+ 2+25*2+4+6 parameter. (C) Historical dynamics of eastern and western groups under p 1+1+1+1+25*2+4+6 parameter. (D) Historical dynamics of each population under p 4+25*2+4+6 parameter. (E) Historical dynamics of each population under p 2+ 2+25*2+4+6 parameter. (F) Historical dynamics of each population under p 1+1+1+1+25*2+4+6 parameter. Shown are PSMC plots depicting the dynamic changes in effective population size of each population represented by different colored lines. The period of the Xixiabangma Glaciation (XG, 0.8-1.17 ka), Naynayxungla Glaciation (NG, 0.5-0.78 ka), and the last glacial maximum (LGM, 12-30 ka) are shaded in grey.

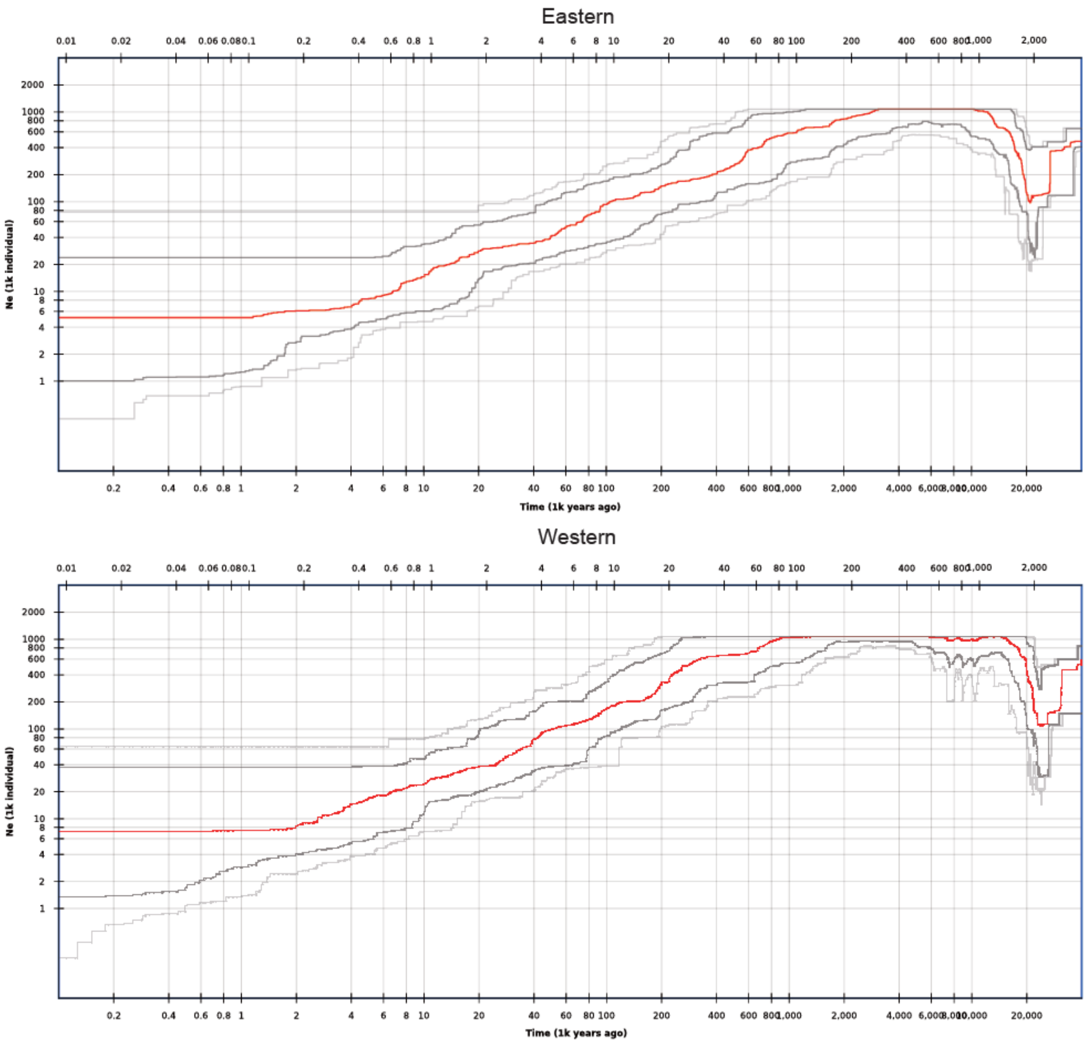

Supplementary Figure 6. Inferred demographic history by Stairway Plot 2 inference with folded SFSs. The solid rad line is median of 200 inferences based on subsampling. Dark gray lines are 75 % confidence interval of the inference. Light gray lines are 95 % confidence interval of the inference.

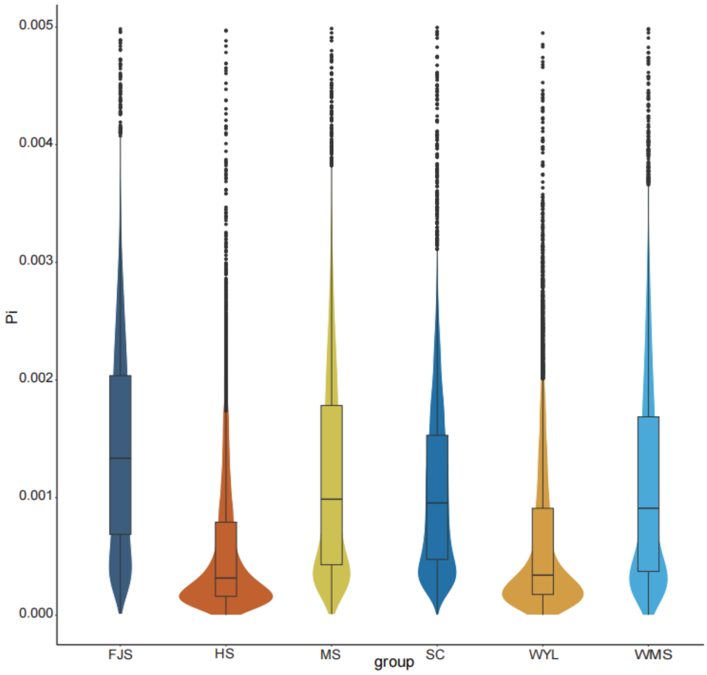

Supplementary Figure 7. Average nucleotide diversity (π) of six populations.

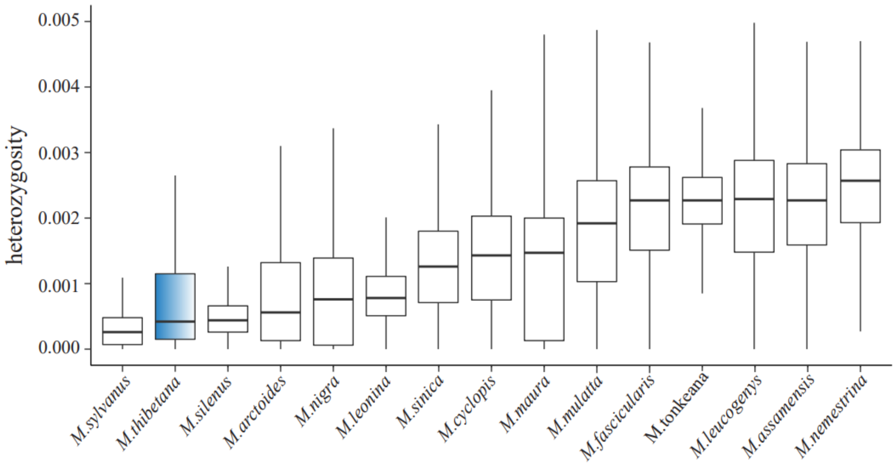

Supplementary Figure 8. Comparison of heterozygosity in 15 species of the genus *Macaca*.

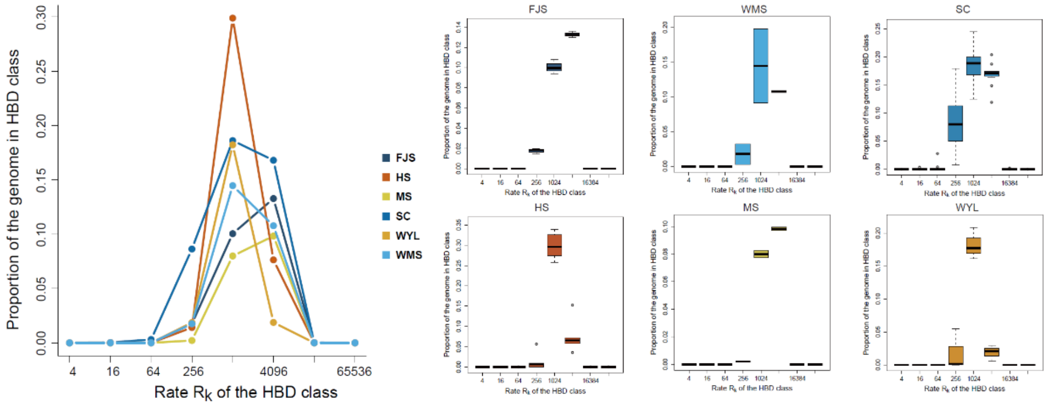

Supplementary Figure 9. The proportion of the genome in HBD class for each population

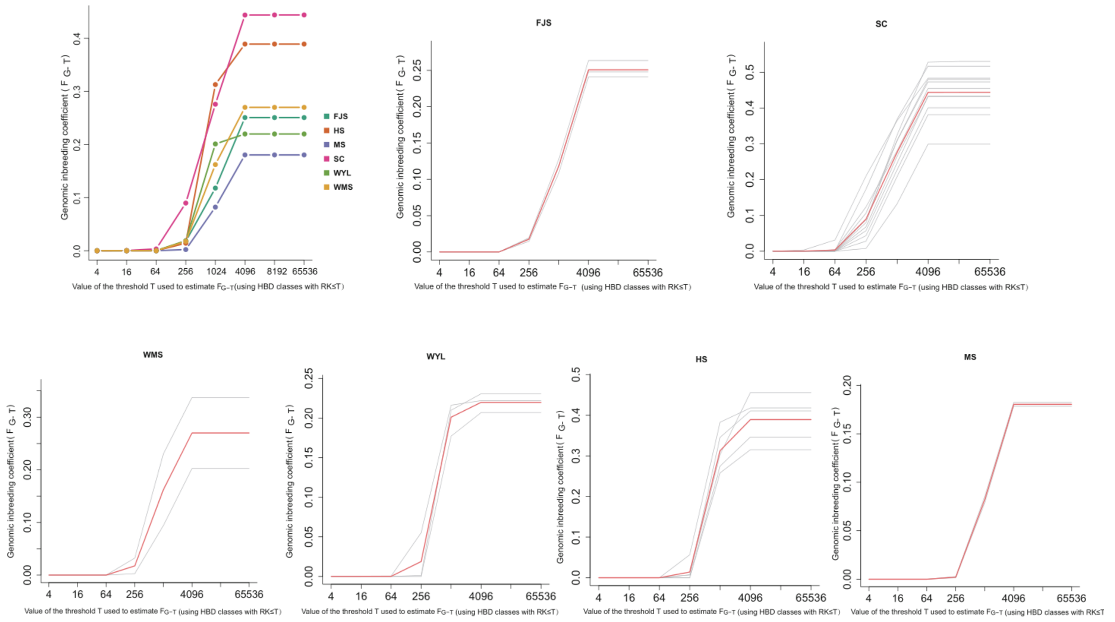

Supplementary Figure 10. The average inbreeding coefficient was estimated using the HBD class. The first figure is the distribution of inbreeding coefficients of 6 populations, and the rest of figure are the distribution of every individual in each population.

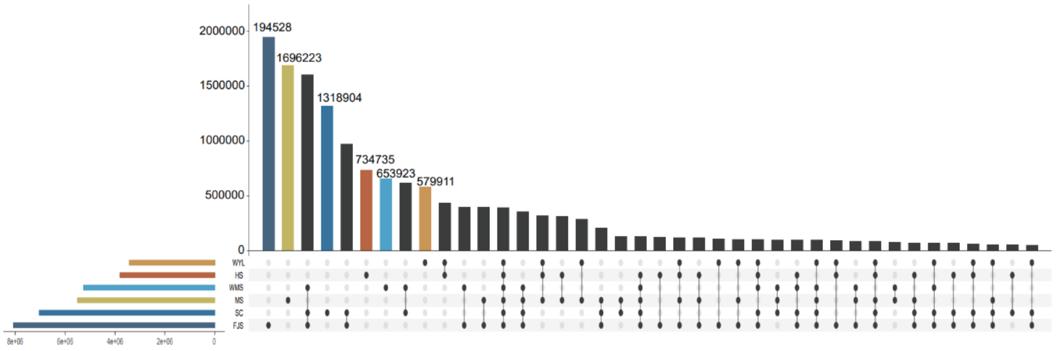

Supplementary Figure 11. The upset plot shows the distribution of shared and unique

SNPs across the six populations. the size of the intersection on the y axis indicates the number of shared SNPs, while the size of the intersection on the x axis indicates the number of unique SNPs.

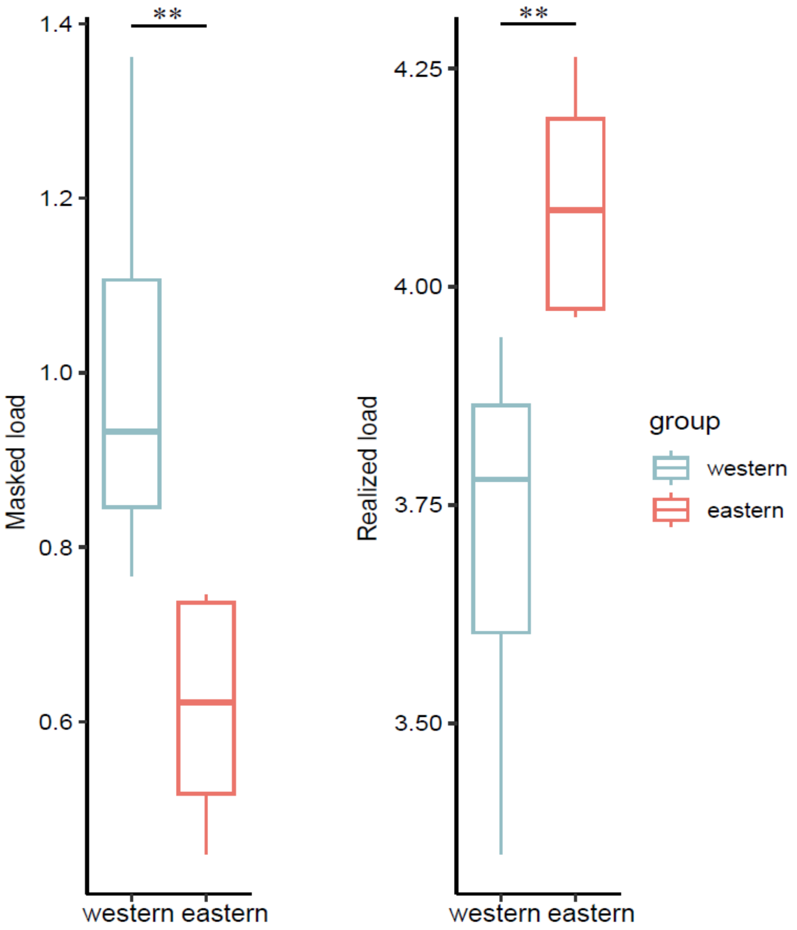

Supplementary Figure 12. Masked load and realized load of the eastern and western groups.

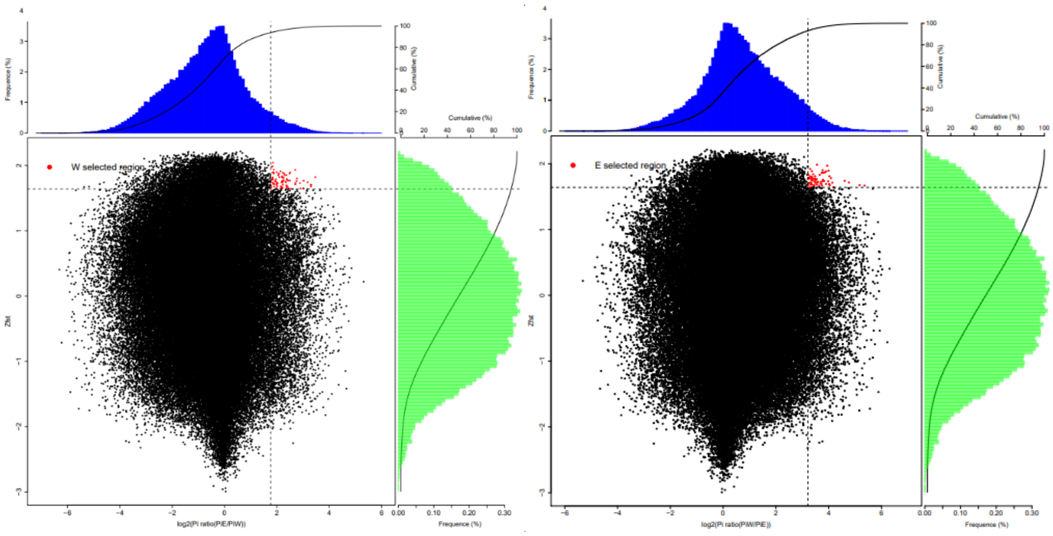

Supplementary Figure 13. Distribution of log2 (θπ ratio (θπ, Eastern/θπ, Western)), (θπ ratio (θπ, Western/θπ, Eastern)) and ZFst values between Eastern and Western populations.

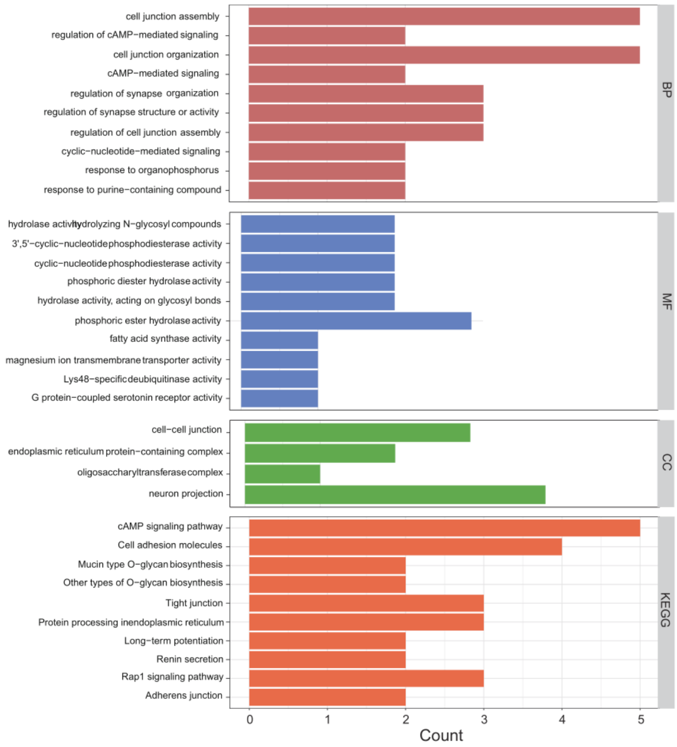

Supplementary Figure 14. Bubble plot for GO and KEGG of 74 overlap candidate genes under adaptive selection identified by XPCLR, CLR, and *Fst*+θπ analysis.

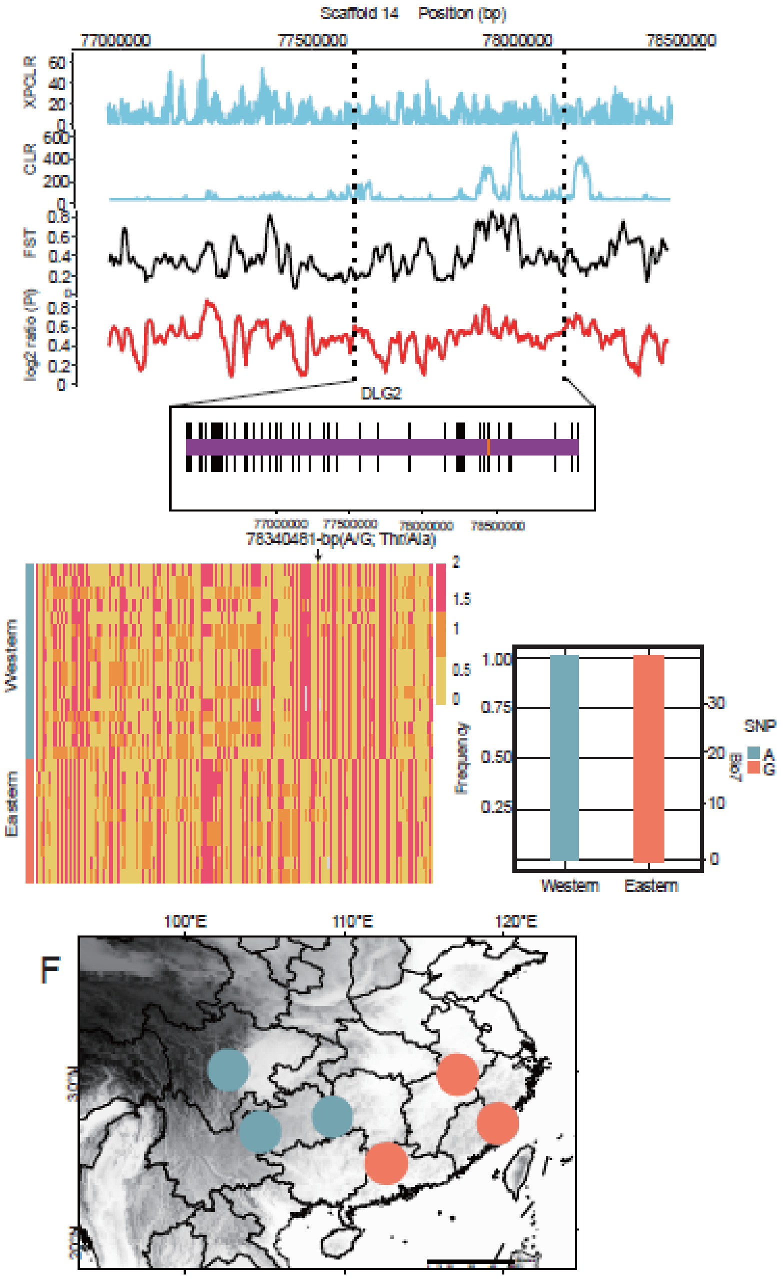

Supplementary Figure 15. Candidate adaptive genes. Signals of the gene region and genetic annotations (black bars refer to coding sequences; all missense variants are shown as orange bars). Genotypes of SNPs, one missense SNP shows different allele frequencies in two BIO7 groups. Allele frequencies reveal patterns of geographic change from western to eastern.

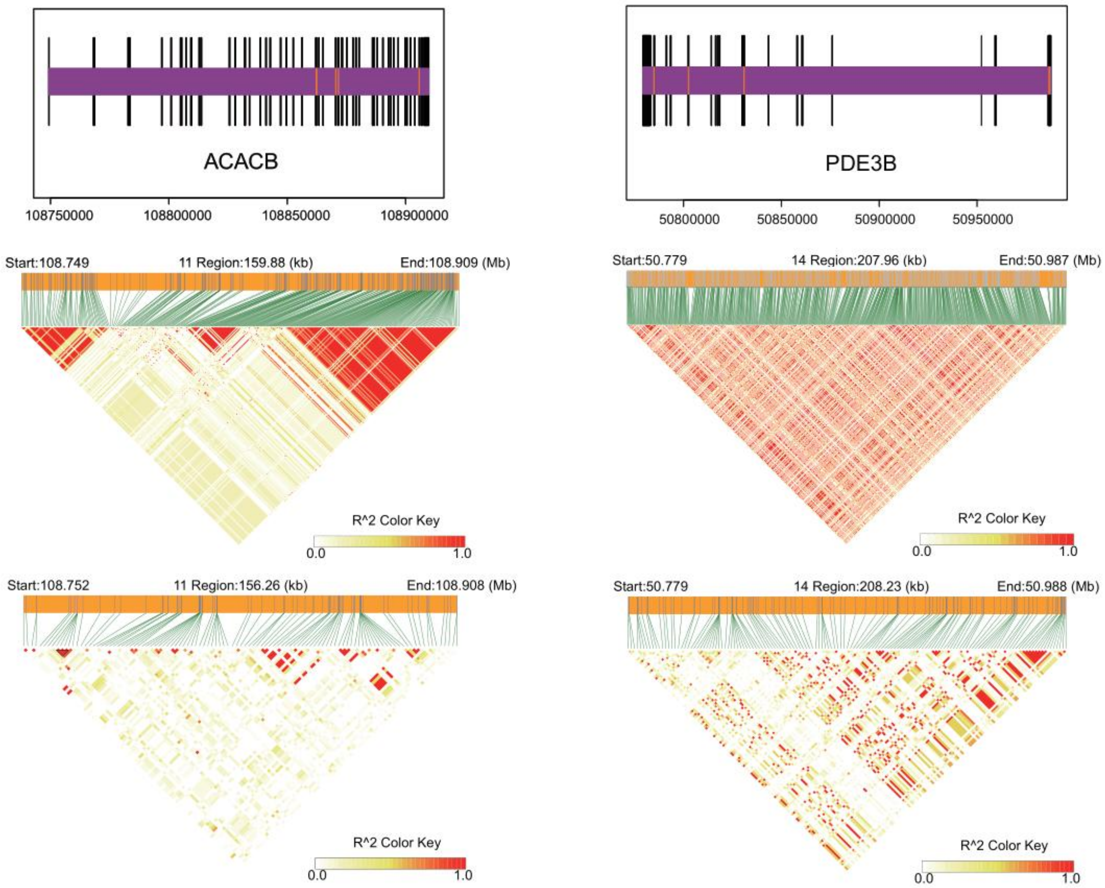

Supplementary Figure 16. LD analyses were performed on the SNPs of *ACACB* and *PDE3B* in the eastern and western populations, respectively. The degree of linkage disequilibrium in this region was based on pairwise D' between SNPs.

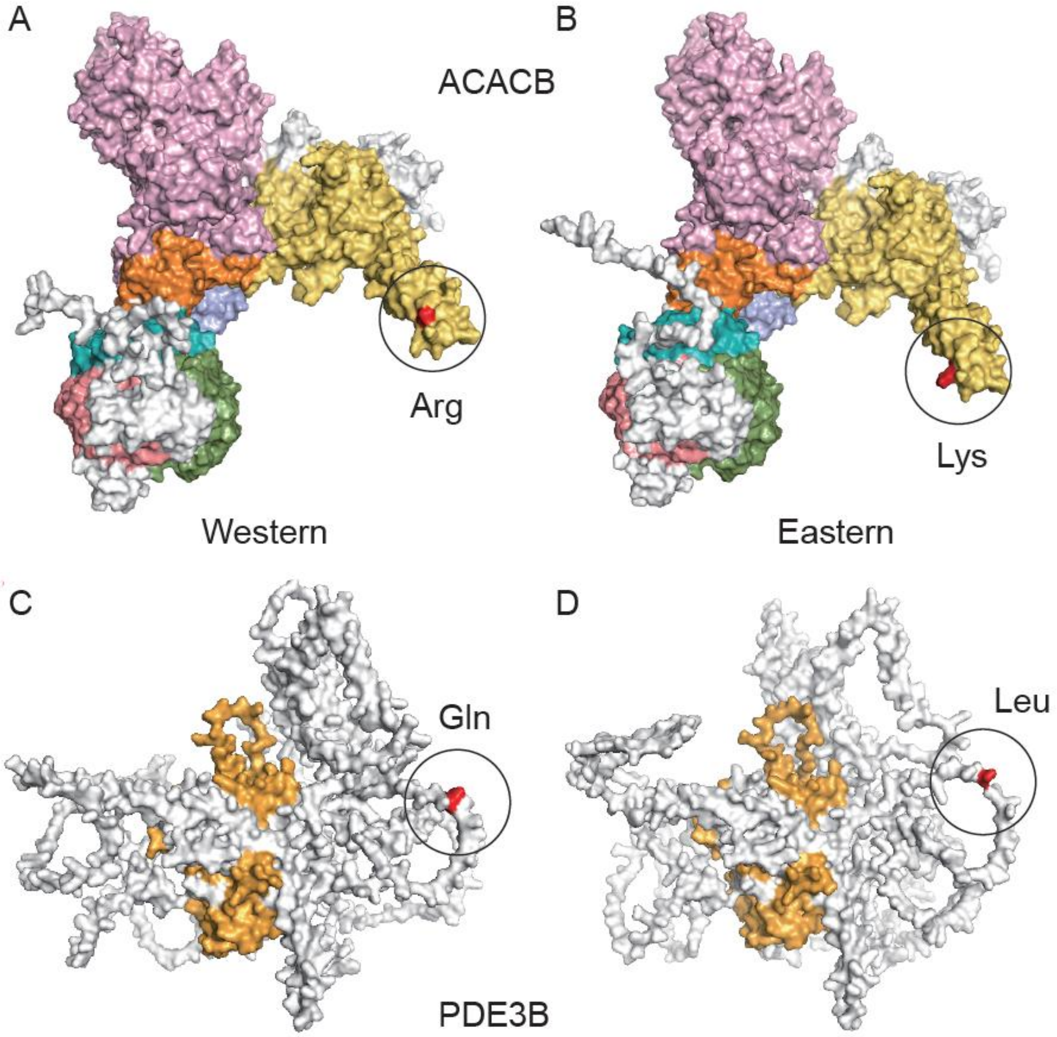

Supplementary Figure 17. Predicted threedimensional structure model of *ACACB* and *PDE3B*. The protein is illustrated in grey surface and missense mutations are indicated in red. (A) and (B) show the predicted threedimensional structure model of *ACACB* in the western and eastern groups, respectively. Carbamoylphosphate synthase L chain, ATP binding domain are shown in samlon color; Biotin carboxylase, Nterminal domain are shown in smudge; Biotinrequiring enzyme are shown in lightblue; Carboxyl transferase domain are shown in yelloworange; AcetylCoA carboxylase, central region are shown in lightpink; Biotin carboxylase Cterminal domain are shown in teal; AcetylCoA carboxylase, BT domain are shown in orange. (C) and (D) show the predicted threedimensional structure model of *PDE3B* in the western and eastern groups, respectively. 3'5'cyclic nucleotide phosphodiesterase domain are shown in brightorange.

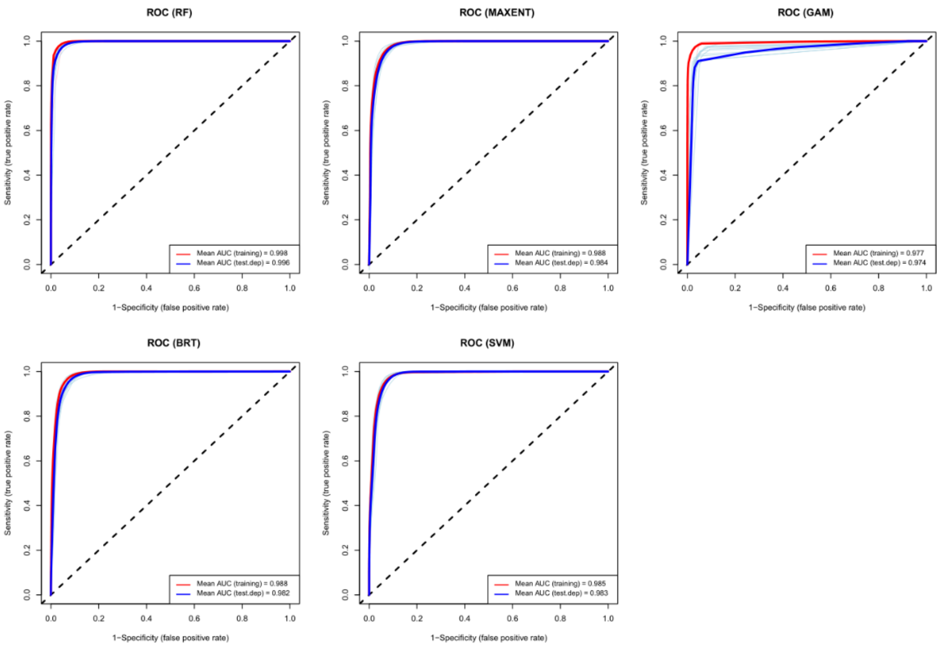

Supplementary Figure 18. Receiver operating characteristic curve (ROC) shows that the models have high accuracy.

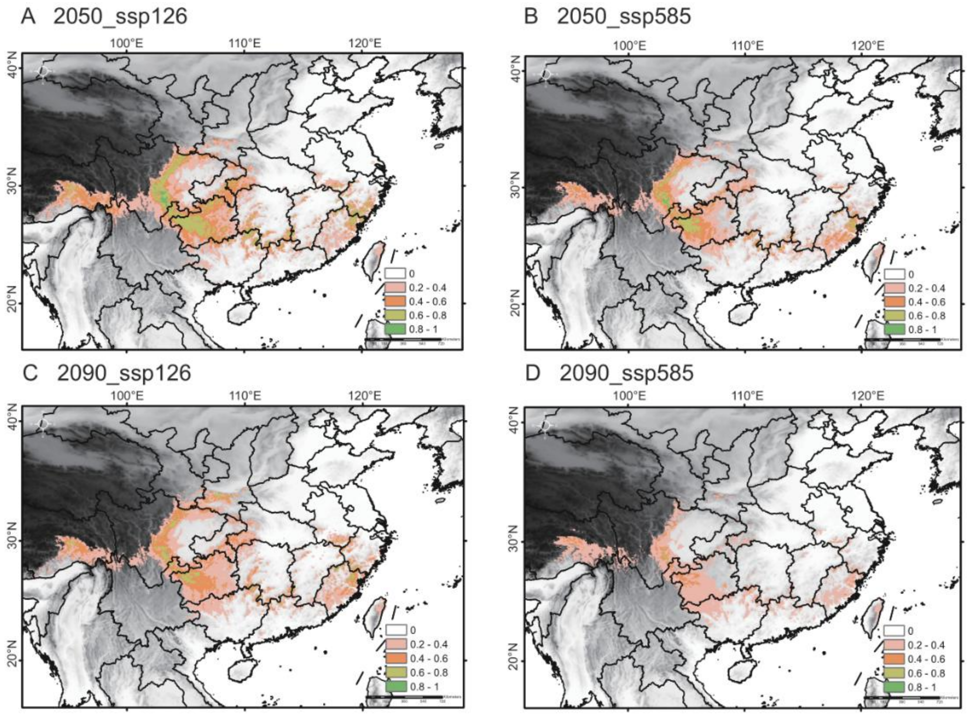

Supplementary Figure 19. Habitat suitability maps for Tibetan macaques in the 2050 SSP 126 scenario and the SSP 585 scenario, and in the 2090 SSP 126 and SSP 585 scenarios.

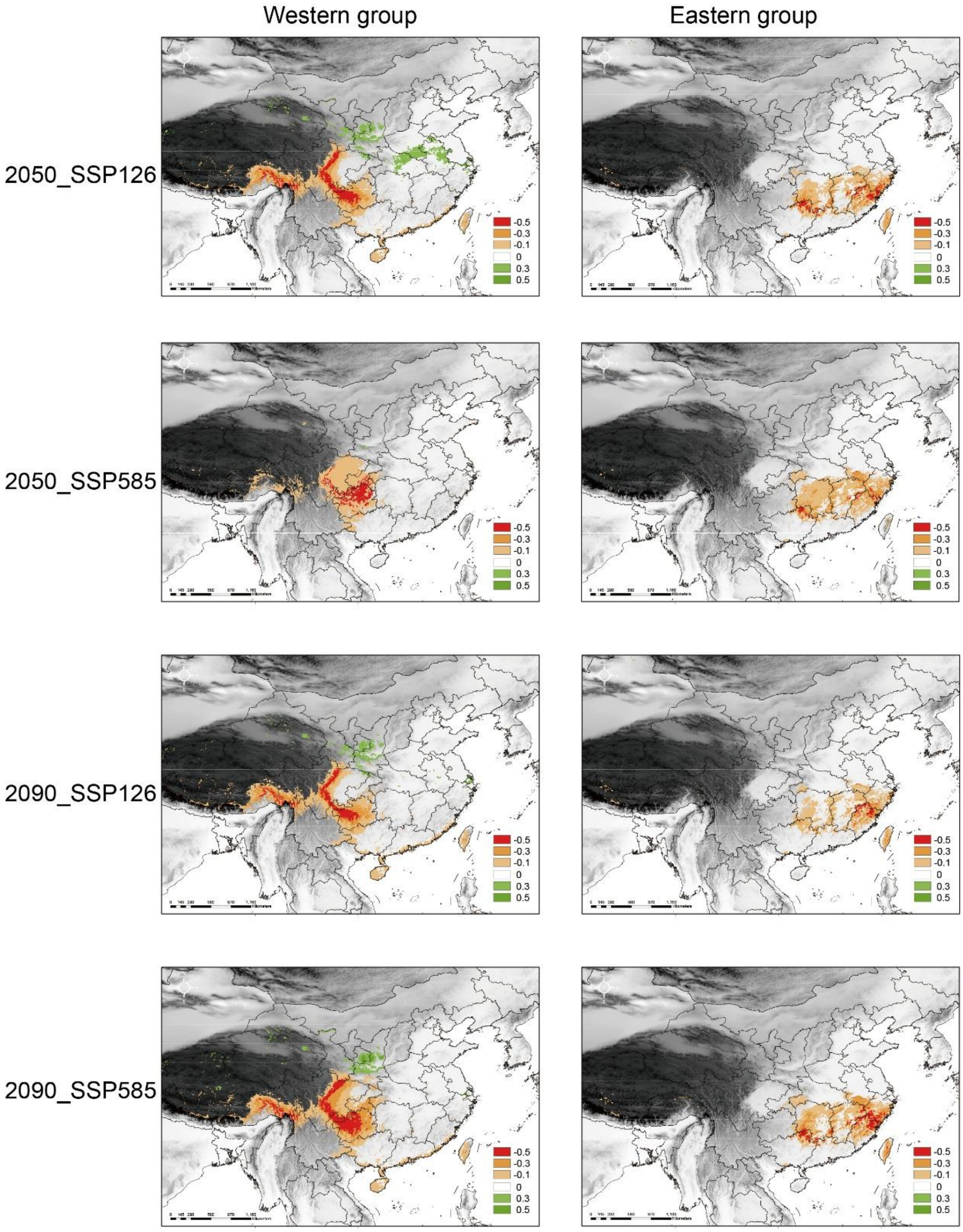

Supplementary Figure 20. Ecological vulnerability maps of the eastern and western Tibetan macaque groups in the 2050 SSP 126 and SSP 585 scenarios and the 2090 SSP 126 and SSP 585 scenarios.

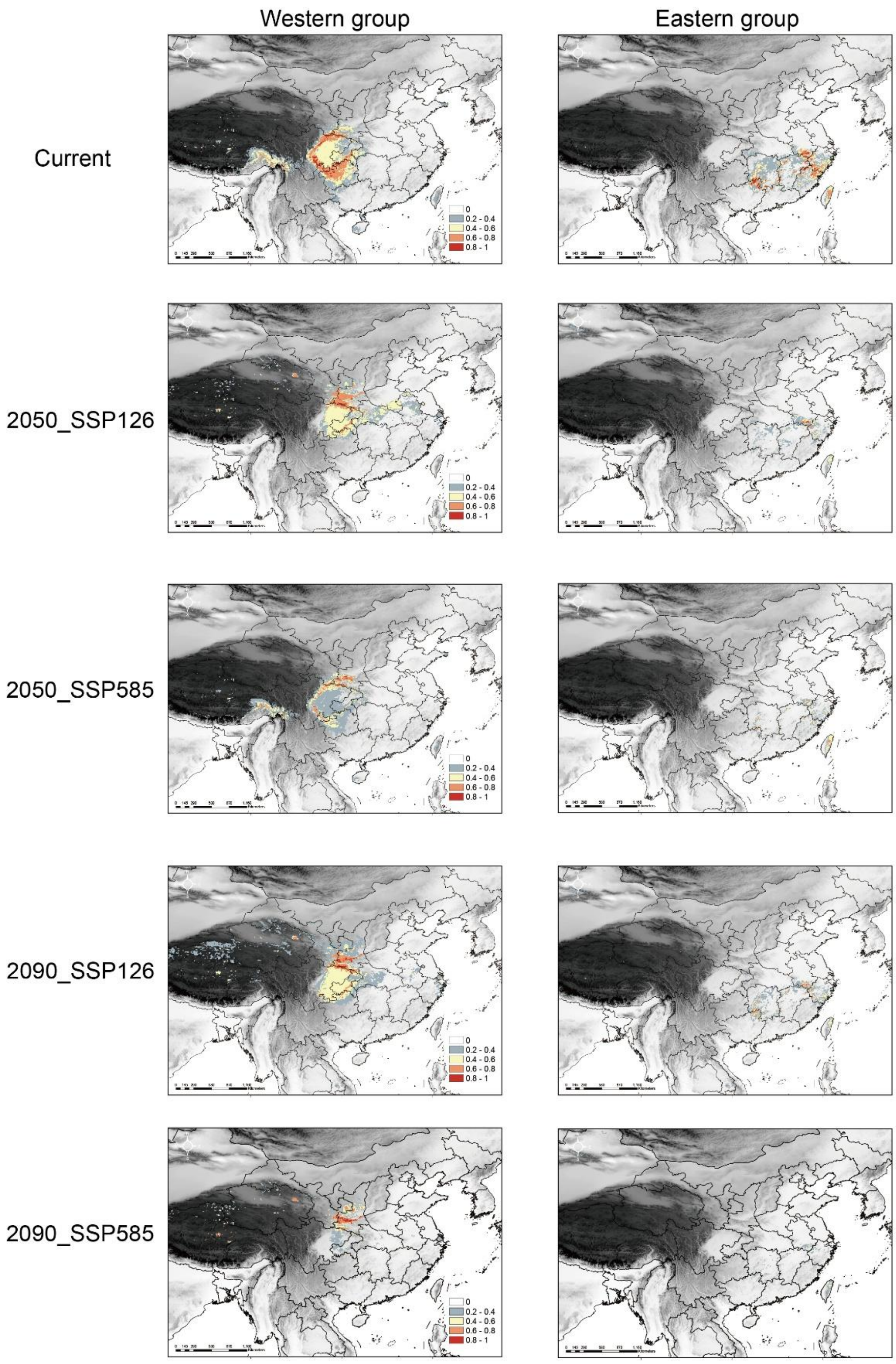

Supplementary Figure 21. Habitat suitability maps of the eastern and western Tibetan macaque groups in the 2050 SSP 126 and SSP 585 scenarios and the 2090 SSP 126 and SSP 585 scenarios.

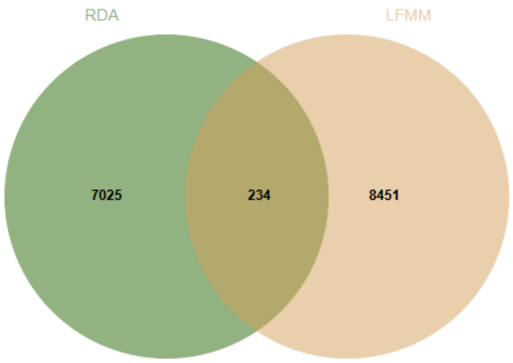

Supplementary Figure 22. Climate-association loci identified using RDA and LFMM.

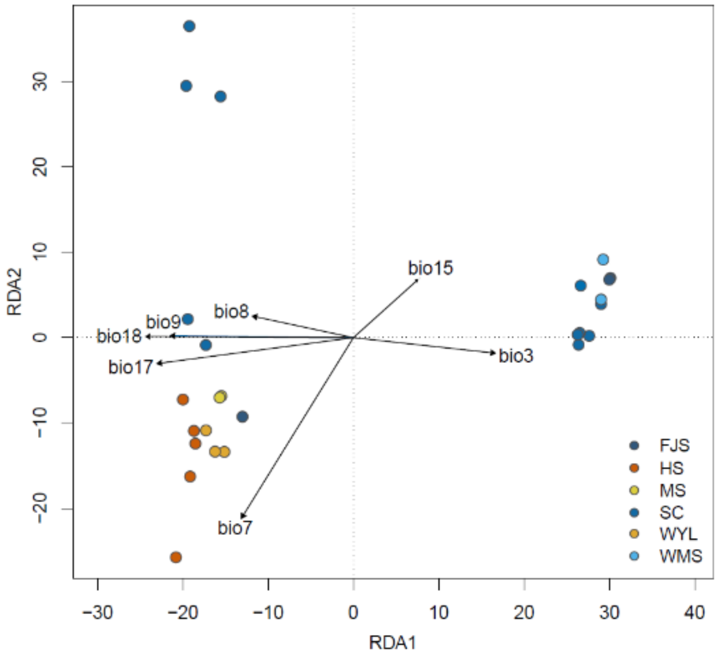

Supplementary Figure 23. PCA plot based on RDA axes 1 and 2. The black arrows present environmental variables. The points present populations.

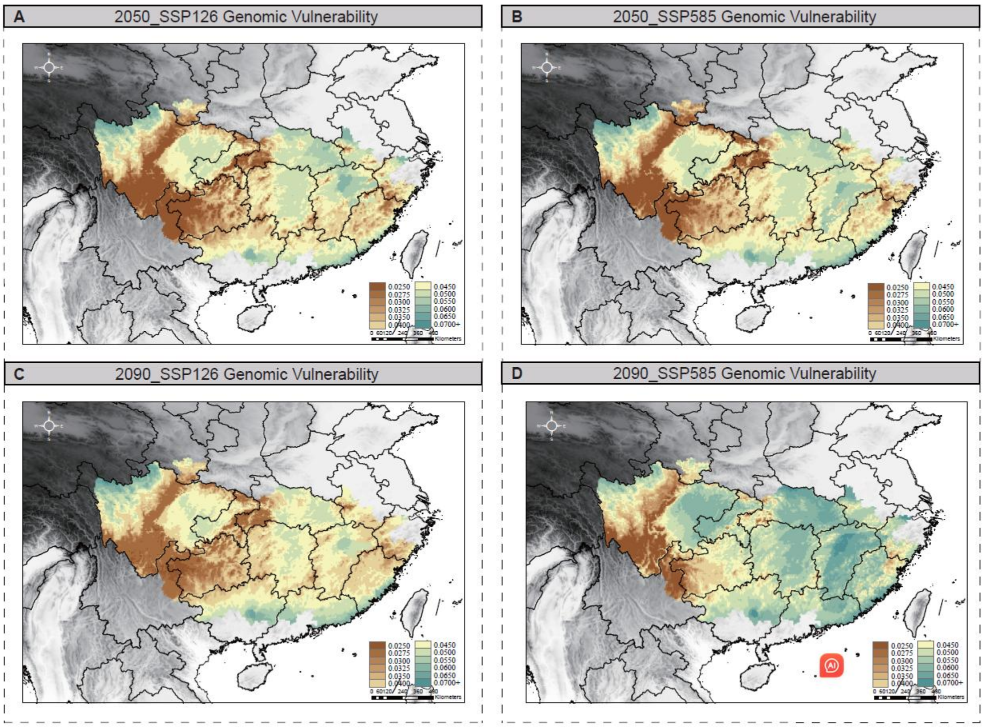

Supplementary Figure 24. GF modeling to calculate genetic offset under different future climate scenarios. The color scale from blue to red refers to the increasing of genetic offset.

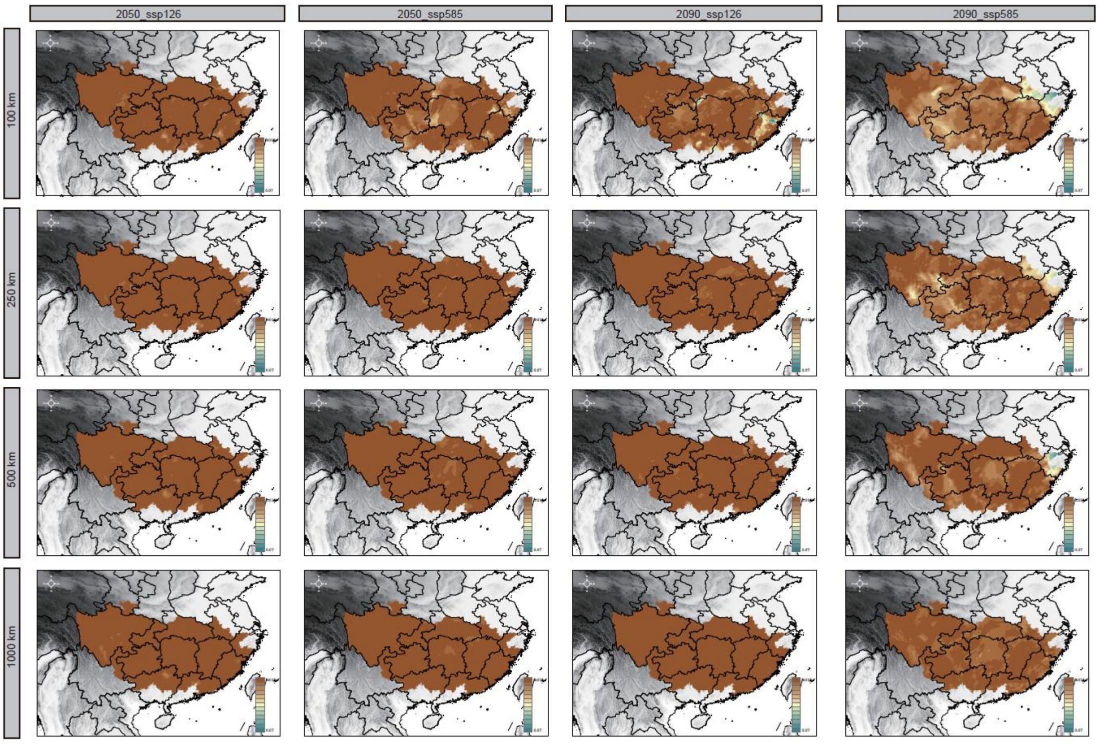

Supplementary Figure 25. Effect of search distance on forward offset from gradient forest. Distance classes included 100 km, 250 km, 500 km, and 1000 km.

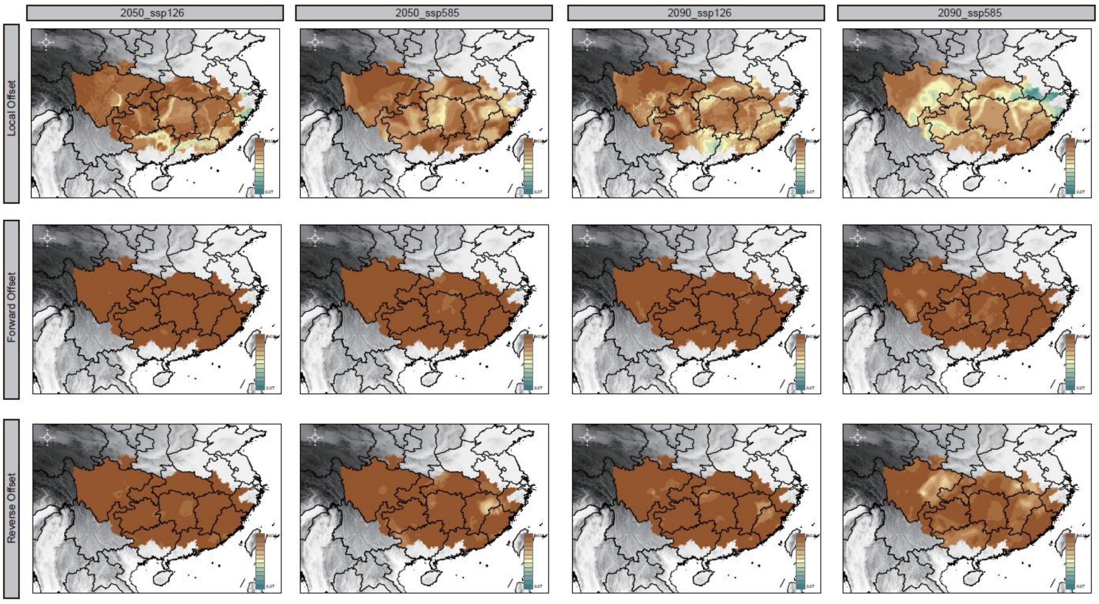

Supplementary Figure 26. GDM modeling to calculate local offset, forward offset, and reverse offset under different future climate scenarios. The color scale from blue to red refers to the increasing of genetic offset.

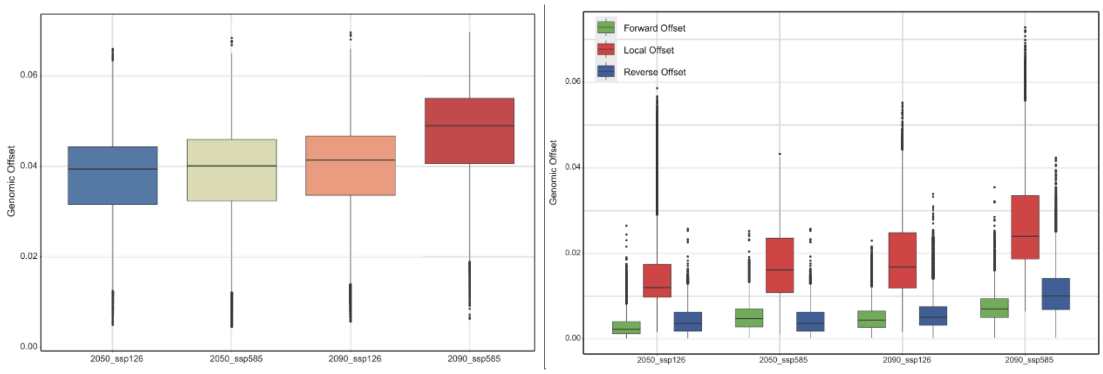

Supplementary Figure 27. The GF and GDM models calculate genomic offset statistics. Genetic offsets progressively increase with increasing climate change.

Supplementary Tables

Supplementary Table S1. Sequencing Statistics

Sample ID	Clean reads	Clean bases (bp)	Mapping rate (%)	Average depth (x)	Sample source	Population
HS1	624504498	93675674700	98.36	28.02	CRR1146567	HS
HS2	639988650	95998297500	98.98%	29.27	CRR1146568	HS
HS3	703499706	105524955900	99.34%	24.54	CRR1146569	HS
JHS	596758964	89513844600	99.41%	24.68	CRR1146571	HS
JD	591076874	88661531100	99.32%	24.14	CRR1146570	HS
WYL1	700686622	105102993300	99.45%	25.46	CRR1146581	WYL
WYL2	590793696	88619054400	99.49%	22.13	CRR1146582	WYL
WYL3	601360728	90204109200	99.44%	22.37	CRR1146583	WYL
MS1	590530048	88579507200	99.35%	23.29	CRR1146577	MS
MS2	601051556	90157733400	99.37%	23.55	CRR1146578	MS
FJS1	635881000	95382150000	99.32%	24.77	CRR1146564	FJS
FJS2	641278810	96191821500	99.51%	22.92	CRR1146565	FJS
FJS3	632119382	94817907300	99.45%	22.72	CRR1146566	FJS
YJ	930186830	139305483572	99.34%	40.3	CRR1146585	WMS
XS	590972328	88645849200	97.92%	22.81	CRR1146584	WMS
BX	633587582	95038137300	99.43%	23.55	CRR1146561	SC
EMS1	694601748	104190262200	99.09%	31.62	CRR1146562	SC
EMS2	612015950	91802392500	99.22%	26.99	CRR1146563	SC
MB1	603511262	90526689300	99.55%	20.45	CRR1146574	SC
MB2	721939922	108834892340	99.09%	32	CRR1146575	SC
MB3	753189644	113542283286	99.06%	33.28	CRR1146576	SC
LBH1	655154016	98273102400	99.19%	29.83	CRR1146572	SC
LBH2	664535889	99680383350	99.07%	30.4	CRR1146573	SC
WC1	644574094	96686114100	99.71%	33.28	CRR1146579	SC
WC2	691349128	103702369200	99.58%	22.74	CRR1146580	SC
BSJ	605922674	90888401100	99.42%	23.95	CRR1146560	SC

Supplementary Table S2. Numerical distribution of SNPs across different genomic regions

Type (alphabetical order)	Count	Percentage (%)
DOWNSTREAM	2,865,899	3.78
EXON	275,154	0.36
INTERGENIC	23,966,075	31.61
INTRON	14,187,137	18.71
SPLICE_SITE_ACCEPTOR	491	0.00
SPLICE_SITE_DONOR	454	0.00
SPLICE_SITE_REGION	24,128	0.03
TRANSCRIPT	88,354,418	41.38
UPSTREAM	2,895,185	3.82
UTR_3_PRIME	199,410	0.26
UTR_5_PRIME	69,374	0.09

Supplementary Table S3. TracyWidom (TW) statistics and pvalues for the ten first eigenvalues in the PCA

Number	Eigenvalue	TWstat	pvalue
1	22.07589	4.206	0.00014
2	5.391962	1.984	0.0106797
3	4.580343	2.05	0.00955867
4	4.178808	3.699	0.000417831
5	3.145328	1.341	0.0296414
6	2.519383	1.715	0.641333
7	2.32755	2.326	0.810107
8	2.02174	4.525	0.998447
9	1.991176	4.154	0.995081
10	1.973514	3.587	0.978696

Supplementary Table S4. Number of different mutations in each population

Population	Homozygous of LoF	Heterozygous of LoF	Synonymous	Missense
FJS	760	75	101543	68273
FJS	729	106	102000	68302
FJS	724	111	103423	68284
HS	846	68	101543	68111
HS	870	49	102000	68125
HS	858	54	103423	68074
HS	885	36	104384	67799
HS	876	35	104384	67657
MS	771	65	101543	68179
MS	769	67	102000	68432
SC	862	58	101543	68375
SC	838	82	102000	67722
SC	768	152	103423	67664
SC	869	51	104384	67555
SC	870	50	104384	68311
SC	778	142	104463	68426
SC	826	94	98844	67656
SC	772	148	100767	67445
SC	854	66	100350	68127
SC	823	97	100114	67413
SC	731	189	103446	67956
WYL	752	95	101543	68556
WYL	801	46	102000	68212
WYL	761	86	103423	68007
WMS	746	89	101543	68151
WMS	790	45	102000	67691

Supplementary Table S5. Scores for 74 genes appearing in at least two methods

Gene_name	Chr	Start_position	End_position	CLR	Xpclr	FST
ACACB	11	108768049	108907914	623.845913	52.2669253	1.663256144
DLG2	14	76618049	78824631	688.298857	64.9114086	1.798244064
PDE3B	14	50782931	50987659	585.789385	63.5089345	1.644387509
ADAMTS6	6	63704589	64032347	681.002458		1.765379774
ESCO1	18	60810422	60891418	717.031782		1.743566791
COG5	3	133516642	133885740	743.828618		1.787051251
MALRD1	9	20185228	20867948	655.527239		1.665248489
GREB1L	18	60897944	61054399	481.599954		1.751164898
CLDN10	17	77217517	77357122	664.536911		1.776437977
EVI5	1	131391649	131675495	595.838869		1.673434593
USP8	7	26829725	26906678	613.029752		1.742134856
POPDC3	4	68045458	68068966	589.435399		1.895436816
KIF9	2	103230959	103279404	615.266504		1.796323591
TRPM3	15	85958515	86539156	367.892426	76.9668842	
SGCB	5	81612244	81629841	464.536431	57.8249678	
GRB14	12	51633262	51761139	400.465874	61.9828013	
AGPAT3	3	2749568	2871242		65.082617	1.674169355
GRIN2A	20	9773628	10193111		59.6420011	1.930614255
NRXN1	13	57712533	58833952		81.1565477	1.695023591
PERM1	1	223442066	223448060		78.1085558	1.689326897
USP18	10	20863382	20898098		175.69227307	1.795626935
XKR4	8	55734648	56145115		58.3800994	1.651476361
MYPN	9	69710819	69828601	442.864431		1.935330165
MARCH6	6	10322185	10405291		64.573262	1.897018394
PREP	4	67815914	67949947	375.835626		1.895969304
TUSC3	8	15649611	15856323		55.8316114	1.884774014
ELOVL6	5	108402791	108551064	464.752154		1.853486151
AQP9	7	34546984	34593785	623.231411		1.83629606
SULT1B1	5	64005689	64040306	576.864569		1.742812142
FOXN4	11	108923383	108952493	602.763552		1.663256144
WDR7	18	23163322	23551308		62.6307906	1.642938766
ANKFN1	16	40306755	40654067	465.522554		1.642708965
ABCC4	17	76792317	77082114		57.5905694	1.896378949

AVL9	3	82539996	82641722		76.2346998	1.662701625
GALNT14	13	77756609	78018185		75.6568675	1.682034856
OPCML	14	125014857	125554082		85.7550245	1.653629493
PARD3	9	35052281	35772961		59.1507973	1.835951359
PKD1L2	20	68129394	68237549		70.1440165	1.759252883
PLXDC2	9	20947660	21406149		73.5965761	1.659724207
PRDM5	5	118540638	118760736		82.4833063	1.719966957
RGS7BP	6	63029671	63137869		56.9312629	1.830571026
ROPN1L	6	10409314	10433279		64.573262	1.787938003
UBE4B	1	214286110	214433669		60.3188909	1.748122536
CCDC141	12	65932953	66190217	359.797022	69.3845373	
CDH4	10	96406502	97092675	542.134369	96.2823418	
ESF1	10	52766075	52836905	492.830146	64.1753349	
ETNK1	11	23950407	24011085	667.9742	56.0933635	
GALNT13	12	40950725	41543398	419.411635	75.9992029	
GLT1D1	11	128708888	128829915		93.9449992	1.753247052
HECTD4	11	111866974	112092296		62.8357118	1.875434022
LINGO2	15	55813387	55815573		58.2356968	1.844212359
MACROD2	10	50459127	52552871	376.243346	56.7637585	
MAST1	19	12412638	12456545		59.3247425	1.612317743
MOB2	14	1525133	1597652		69.7030601	1722884415
NAA25	11	111721104	111804689		57.6229926	1.834211195
NAV3	11	77410118	77790695	347.911676	61.0386104	
NBEA	17	13857743	14598476	670.322602	63.3654059	
NCKAP5	12	17856283	18640562		56.7362107	1.722178834
NUMA1	14	64949504	65031706		57.6018681	1.793562195
PPP6R2	10	1046612	1098862	390.350723	61.0192453	
PTPRD	15	75019406	75438879		78.8511153	1.624672252
PTPRO	11	16733014	17001802		58.2678368	1.723121424
RAP1B	11	68145962	68196880	354.571523	57.3639914	
RBFOX1	20	6110059	7799741	471.559242	65.3205717	
RNF169	14	67798320	67889884		55.7844716	1.852119341
SEL1L2	10	52462895	52696062	422.852639	57.2049092	
SNX29	20	11951037	12535282		88.2986085	1.753214934
SREBF2	10	9599445	9679358	375.242226	59.2336144	

THSD7B	12	21938378	22868030	474.153581	55.8376145	
USP7	20	8917099	8989213	413.742174	60.6994239	
XRRA1	14	67896137	67999615	421.986519	62.4385283	
MAP2	12	96758711	97070714	634.987699		1.778246409
PLA2R1	12	46986014	47110781	613.75319		1.745379915
ESRRG	1	73998664	74592891		61.1890483	1.673632422

Supplementary Table S6. Environmental variables used in this study derived form WorldClim

**Climatic variables**	**Definition**
BIO1	Annual mean temperature
BIO2	Mean diurnal range
BIO3	Isothermality (BIO2/BIO7)
BIO4	Temperature Seasonality
BIO5	Maximum Temperature of Warmest Month
BIO6	Minimum Temperature of Coldest Month
BIO7	Temperature Annual Range (BIO5BIO6)
BIO8	Mean Temperature of Wettest Quarter
BIO9	Mean Temperature of Driest Quarter
BIO10	Mean Temperature of Warmest Quarter
BIO11	Mean Temperature of Coldest Quarter
BIO12	Annual Precipitation
BIO13	Precipitation of Wettest Month
BIO14	Precipitation of Driest Month
BIO15	Precipitation Seasonality
BIO16	Precipitation of Wettest Quarter
BIO17	Precipitation of Driest Quarter
BIO18	Precipitation of Warmest Quarter
BIO19	Precipitation of Coldest Quarter

Supplementary Table S7. Summarizing the low and high groups for the selection signature analyses

**Environmental Variables**	**Low Group Populations and Environmental Stat (blue dots)**	**High Group Populations and Environmental Stat (red dots)**	**Locations on the Map**
**Temperature Annual Range (BIO7)**	Sichuan, Yunnan (n=8)<br>Mean BIO7: 25.921<br>Min T(°C): 24.904<br>Max T(°C): 26.883	Anhui, Zhejiang (n=8)<br>Mean BIO7: 29.048<br>Min T(°C): 27.179<br>Max T(°C): 31.247	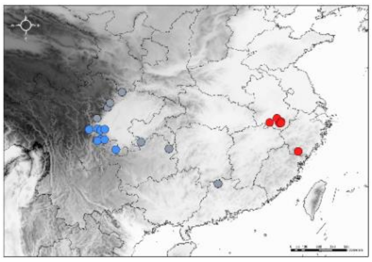

Supplementary Table S8. Variable inflation factors (VIFs) of the remaining variables for SDMs

**Variables**	**VIF**
Bio3	3.896870
Bio7	1.793945
bio8	6.036571
bio9	8.210976
bio15	5.383504
Bio17	4.498731
bio18	1.912649

Supplementary Table S9. Model mean performance

**Methods**	**AUC**	**TSS**
RF (random forests)	0.99	0.96
MAXENT (maximum entropy)	0.98	0.91
GAM (generalized additive model)	0.97	0.93
BRT (boosted regression trees)	0.98	0.91
SVM (support vector machine)	0.98	0.91

Supplementary Table S10. Ecological vulnerability values of various populations of Tibetan macaques under different emission scenarios

**Population**	**2050_SSP126**	**2050_SSP585**	**2090_SSP126**	**2090_SSP585**
HS	0.27529377	0.23736346	0.49374941	0.456177729
WYL	0.140651743	0.371667316	0.429546793	0.498311818
MS	0.018898487	0.052821968	0.059110593	0.063098731
FJS	0.062420011	0.200898141	0.254531026	0.40145972
WMS	0.29995051	0.350591108	0.0332708106	0.593703419
SC	0.099834136	0.229653629	0.239226287	0.406662732

Supplementary Table S11. Importance of components in the RDA

**Constrained axes**	RDA1	RDA2	RDA3	RDA4	RDA5
**Eigenvalue**	131839	43694	33534	31871	24984
**Proportion explained**	0.4958	0.1643	0.1261	0.1198	0.9395
**Cumulative proportion**	0.4958	0.6601	0.7862	0.9060	1.0000

Supplementary Table S12. The environmental associated SNPs and the loading, correlation with each predictor from RDA (top 1% of data shown)

Position	loading	bio7	bio8	bio9	bio15	bio18	predictor	correlation
Chr1.142709733	0.02276	0.119637	0.79913	0.87451	0.429862	0.68062	bio9	0.874507
Chr5.79576806	0.02276	0.119637	0.79913	0.87451	0.429862	0.68062	bio9	0.874507
Chr5.162311519	0.02276	0.119637	0.79913	0.87451	0.429862	0.68062	bio9	0.874507
Chr6.16980832	0.02276	0.119637	0.79913	0.87451	0.429862	0.68062	bio9	0.874507
Chr6.165199529	0.02276	0.119637	0.79913	0.87451	0.429862	0.68062	bio9	0.874507
Chr6.170444279	0.02276	0.119637	0.79913	0.87451	0.429862	0.68062	bio9	0.874507
Chr7.23774186	0.02276	0.119637	0.79913	0.87451	0.429862	0.68062	bio9	0.874507
Chr7.68515289	0.02276	0.119637	0.79913	0.87451	0.429862	0.68062	bio9	0.874507
Chr8.73700351	0.02276	0.119637	0.79913	0.87451	0.429862	0.68062	bio9	0.874507
Chr8.98035031	0.02276	0.119637	0.79913	0.87451	0.429862	0.68062	bio9	0.874507
Chr10.30609751	0.02276	0.119637	0.79913	0.87451	0.429862	0.68062	bio9	0.874507
Chr10.30609756	0.02276	0.119637	0.79913	0.87451	0.429862	0.68062	bio9	0.874507
Chr11.25292711	0.02276	0.119637	0.79913	0.87451	0.429862	0.68062	bio9	0.874507
Chr11.79042291	0.02276	0.119637	0.79913	0.87451	0.429862	0.68062	bio9	0.874507
Chr12.20675081	0.02276	0.119637	0.79913	0.87451	0.429862	0.68062	bio9	0.874507
Chr12.22016512	0.02276	0.119637	0.79913	0.87451	0.429862	0.68062	bio9	0.874507
Chr12.88193766	0.02276	0.119637	0.79913	0.87451	0.429862	0.68062	bio9	0.874507
Chr13.5498566	0.02276	0.119637	0.79913	0.87451	0.429862	0.68062	bio9	0.874507
Chr13.82415838	0.02276	0.119637	0.79913	0.87451	0.429862	0.68062	bio9	0.874507

Chr17.21080919	0.02276	0.119637	0.79913	0.87451	0.429862	0.68062	bio9	0.874507
Chr19.8440785	0.02276	0.119637	0.79913	0.87451	0.429862	0.68062	bio9	0.874507
Chr19.40111421	0.02276	0.119637	0.79913	0.87451	0.429862	0.68062	bio9	0.874507
Chr19.40111455	0.02276	0.119637	0.79913	0.87451	0.429862	0.68062	bio9	0.874507
Chr1.17357728	0.02371	0.05738	0.8684	0.8486	0.367618	0.54255	bio8	0.868396
Chr1.36160153	0.02371	0.05738	0.8684	0.8486	0.367618	0.54255	bio8	0.868396
Chr1.66137741	0.02371	0.05738	0.8684	0.8486	0.367618	0.54255	bio8	0.868396
Chr1.156319221	0.02371	0.05738	0.8684	0.8486	0.367618	0.54255	bio8	0.868396
Chr1.172533326	0.02371	0.05738	0.8684	0.8486	0.367618	0.54255	bio8	0.868396
Chr1.173635434	0.02371	0.05738	0.8684	0.8486	0.367618	0.54255	bio8	0.868396
Chr1.213371141	0.02371	0.05738	0.8684	0.8486	0.367618	0.54255	bio8	0.868396
Chr2.60846523	0.02371	0.05738	0.8684	0.8486	0.367618	0.54255	bio8	0.868396
Chr2.106820001	0.02371	0.05738	0.8684	0.8486	0.367618	0.54255	bio8	0.868396
Chr2.136119468	0.02371	0.05738	0.8684	0.8486	0.367618	0.54255	bio8	0.868396
Chr2.136119472	0.02371	0.05738	0.8684	0.8486	0.367618	0.54255	bio8	0.868396
Chr2.140966959	0.02371	0.05738	0.8684	0.8486	0.367618	0.54255	bio8	0.868396
Chr3.574270	0.02371	0.05738	0.8684	0.8486	0.367618	0.54255	bio8	0.868396
Chr3.16159122	0.02371	0.05738	0.8684	0.8486	0.367618	0.54255	bio8	0.868396
Chr3.34588110	0.02371	0.05738	0.8684	0.8486	0.367618	0.54255	bio8	0.868396
Chr3.37725259	0.02371	0.05738	0.8684	0.8486	0.367618	0.54255	bio8	0.868396
Chr3.45501165	0.02371	0.05738	0.8684	0.8486	0.367618	0.54255	bio8	0.868396
Chr3.53220757	0.02371	0.05738	0.8684	0.8486	0.367618	0.54255	bio8	0.868396

Chr3.5514<br>1281	0.02371	0.05738	0.868396	0.848603	0.36762	0.54254<br>6	bio8	0.868396
Chr3.7227<br>5018	0.02371	0.05738	0.8684	0.8486	0.367618	0.54255	bio8	0.868396
Chr3.9104<br>8708	0.02371	0.05738	0.8684	0.8486	0.367618	0.54255	bio8	0.868396
Chr3.1123<br>13894	0.02371	0.05738	0.8684	0.8486	0.367618	0.54255	bio8	0.868396
Chr3.1137<br>25560	0.02371	0.05738	0.8684	0.8486	0.367618	0.54255	bio8	0.868396
Chr3.1284<br>19184	0.02371	0.05738	0.8684	0.8486	0.367618	0.54255	bio8	0.868396
Chr3.1575<br>89512	0.02371	0.05738	0.8684	0.8486	0.367618	0.54255	bio8	0.868396
Chr3.1697<br>23332	0.02371	0.05738	0.8684	0.8486	0.367618	0.54255	bio8	0.868396
Chr3.1762<br>09336	0.02371	0.05738	0.8684	0.8486	0.367618	0.54255	bio8	0.868396
Chr4.2716<br>9467	0.02371	0.05738	0.8684	0.8486	0.367618	0.54255	bio8	0.868396
Chr4.1214<br>05715	0.02371	0.05738	0.8684	0.8486	0.367618	0.54255	bio8	0.868396
Chr4.1593<br>43368	0.02371	0.05738	0.8684	0.8486	0.367618	0.54255	bio8	0.868396
Chr5.7630<br>80	0.02371	0.05738	0.8684	0.8486	0.367618	0.54255	bio8	0.868396
Chr5.6375<br>281	0.02371	0.05738	0.8684	0.8486	0.367618	0.54255	bio8	0.868396
Chr5.7481<br>642	0.02371	0.05738	0.8684	0.8486	0.367618	0.54255	bio8	0.868396
Chr5.3916<br>3448	0.02371	0.05738	0.8684	0.8486	0.367618	0.54255	bio8	0.868396
Chr5.4536<br>8148	0.02371	0.05738	0.8684	0.8486	0.367618	0.54255	bio8	0.868396
Chr5.1153<br>19993	0.02371	0.05738	0.8684	0.8486	0.367618	0.54255	bio8	0.868396
Chr6.1756<br>6386	0.02371	0.05738	0.8684	0.8486	0.367618	0.54255	bio8	0.868396
Chr6.4810<br>2039	0.02371	0.05738	0.8684	0.8486	0.367618	0.54255	bio8	0.868396
Chr6.4810<br>2048	0.02371	0.05738	0.8684	0.8486	0.367618	0.54255	bio8	0.868396
Chr6.1203<br>72596	0.02371	0.05738	0.8684	0.8486	0.367618	0.54255	bio8	0.868396

Chr6.134165541	0.02371	0.05738	0.8684	0.8486	0.367618	0.54255	bio8	0.868396
Chr6.165140013	0.02371	0.05738	0.8684	0.8486	0.367618	0.54255	bio8	0.868396
Chr6.172380762	0.02371	0.05738	0.8684	0.8486	0.367618	0.54255	bio8	0.868396
Chr7.6937045	0.02371	0.05738	0.8684	0.8486	0.367618	0.54255	bio8	0.868396
Chr7.41030337	0.02371	0.05738	0.8684	0.8486	0.367618	0.54255	bio8	0.868396
Chr7.45238777	0.02371	0.05738	0.8684	0.8486	0.367618	0.54255	bio8	0.868396
Chr7.45238783	0.02371	0.05738	0.8684	0.8486	0.367618	0.54255	bio8	0.868396
Chr7.77817644	0.02371	0.05738	0.8684	0.8486	0.367618	0.54255	bio8	0.868396
Chr7.107440655	0.02371	0.05738	0.8684	0.8486	0.367618	0.54255	bio8	0.868396
Chr7.122793221	0.02371	0.05738	0.8684	0.8486	0.367618	0.54255	bio8	0.868396
Chr8.35610834	0.02371	0.05738	0.8684	0.8486	0.367618	0.54255	bio8	0.868396
Chr8.101079614	0.02371	0.05738	0.8684	0.8486	0.367618	0.54255	bio8	0.868396
Chr8.128166886	0.02371	0.05738	0.8684	0.8486	0.367618	0.54255	bio8	0.868396
Chr8.144463407	0.02371	0.05738	0.8684	0.8486	0.367618	0.54255	bio8	0.868396

Supplementary Table S13. SNPs screened from LFMM (top 1% of data shown)

**Position**	**p-values**	**FDR q-value**
Chr15.10269724	1.87E-12	2.29E-05
Chr15.5082590	5.02E-12	2.29E-05
Chr20.12611823	5.02E-12	2.29E-05
Chr3.65432815	1.38E-11	4.71E-05
Chr18.13219271	3.25E-11	8.88E-05
Chr19.54008721	7.16E-11	0.000163123
Chr3.139916180	9.64E-11	0.000188073
Chr10.38416323	1.24E-10	0.000212469
Chr3.139915320	2.29E-10	0.000223683
Chr3.139915400	2.29E-10	0.000223683
Chr3.139916370	2.29E-10	0.000223683
Chr3.139917193	2.29E-10	0.000223683
Chr3.139917382	2.29E-10	0.000223683
Chr3.139922397	2.29E-10	0.000223683
Chr5.179084490	4.17E-10	0.000379505
Chr20.11187361	5.58E-10	0.000426971
Chr14.15957201	5.80E-10	0.000426971
Chr14.15958317	5.80E-10	0.000426971
Chr14.15957523	5.94E-10	0.000426971
Chr2.18751674	9.68E-10	0.000524115
Chr1.197661932	1.05E-09	0.000524115
Chr1.9662231	1.42E-09	0.000524115
Chr1.9667453	1.42E-09	0.000524115
Chr1.88505512	1.42E-09	0.000524115
Chr2.128255627	1.42E-09	0.000524115
Chr2.128256057	1.42E-09	0.000524115
Chr2.128256389	1.42E-09	0.000524115
Chr2.128256813	1.42E-09	0.000524115
Chr2.128256833	1.42E-09	0.000524115
Chr2.128257151	1.42E-09	0.000524115
Chr2.128257878	1.42E-09	0.000524115
Chr2.164474819	1.42E-09	0.000524115
Chr2.164475500	1.42E-09	0.000524115

Chr2.164478177	1.42E-09	0.000524115
Chr2.164481546	1.42E-09	0.000524115
Chr5.5041651	1.42E-09	0.000524115
Chr19.11954344	1.42E-09	0.000524115
Chr3.43638290	1.54E-09	0.000554174
Chr13.72029691	1.95E-09	0.000684281
Chr2.14658318	2.46E-09	0.000730509
Chr2.14658319	2.46E-09	0.000730509
Chr2.14658749	2.46E-09	0.000730509
Chr2.14660618	2.46E-09	0.000730509
Chr2.14660705	2.46E-09	0.000730509
Chr2.14660706	2.46E-09	0.000730509
Chr2.14661046	2.46E-09	0.000730509
Chr1.129117242	3.35E-09	0.00097395
Chr4.126124862	3.71E-09	0.00104826
Chr7.160145189	4.45E-09	0.00104826
Chr7.160146308	4.45E-09	0.00104826
Chr7.160147812	4.45E-09	0.00104826
Chr7.160149593	4.45E-09	0.00104826
Chr7.160149807	4.45E-09	0.00104826
Chr7.160150895	4.45E-09	0.00104826
Chr7.160152046	4.45E-09	0.00104826
Chr7.160152088	4.45E-09	0.00104826
Chr7.160152373	4.45E-09	0.00104826
Chr7.160153551	4.45E-09	0.00104826
Chr14.122189664	5.01E-09	0.00110313
Chr14.122200344	5.01E-09	0.00110313
Chr14.122238402	5.01E-09	0.00110313
Chr14.122200130	5.01E-09	0.00110313
Chr3.139915133	5.13E-09	0.001104919
Chr16.10413209	5.18E-09	0.001104919
Chr10.21880753	6.08E-09	0.001279036
Chr6.151518461	6.45E-09	0.00133471
Chr8.120206908	6.77E-09	0.001380336
Chr16.11302833	7.07E-09	0.00142109

Chr2.97967162	8.06E-09	0.001551327
Chr2.97967485	8.06E-09	0.001551327
Chr2.97968024	8.06E-09	0.001551327
Chr10.40135738	8.28E-09	0.001571765
Chr18.13241524	8.73E-09	0.001634377
Chr3.97857467	9.22E-09	0.001680027
Chr3.97864801	9.22E-09	0.001680027
Chr1.221165040	1.25E-08	0.002248988
Chr3.55205002	1.82E-08	0.003140215
Chr10.98721968	1.90E-08	0.003140215
Chr10.98738531	1.90E-08	0.003140215
Chr10.98738755	1.90E-08	0.003140215
Chr10.98743088	1.90E-08	0.003140215
Chr6.148246102	1.91E-08	0.003140215
Chr6.148253296	1.91E-08	0.003140215
Chr2.2902657	1.98E-08	0.003228174
Chr20.2679593	2.17E-08	0.003362273
Chr4.24168622	2.27E-08	0.003362273
Chr10.21847620	2.58E-08	0.003362273
Chr10.21849397	2.58E-08	0.003362273
Chr10.21849419	2.58E-08	0.003362273
